# Higher-order organisation of extremely amplified, potentially functional and massively methylated 5S rDNA in European pikes (*Esox* sp.)

**DOI:** 10.1186/s12864-017-3774-7

**Published:** 2017-05-18

**Authors:** Radka Symonová, Konrad Ocalewicz, Lech Kirtiklis, Giovanni Battista Delmastro, Šárka Pelikánová, Sonia Garcia, Aleš Kovařík

**Affiliations:** 1 Research Institute for Limnology, University of Innsbruck, Mondseestrasse 9, A-5310 Mondsee, Austria; 2 Department of Marine Biology and Ecology, Faculty of Oceanography and Geography, University of Gdansk, Av. Marszałka Piłsudskiego 46, Gdynia, 81-378 Poland; 3 Department of Ichthyology, Faculty of Environmental Sciences, University of Warmia and Mazury, M. Oczapowskiego Str. 5, 10-718 Olsztyn, Poland; 4 Department of Zoology, Faculty of Biology and Biotechnology, University of Warmia and Mazury, M. Oczapowskiego Str. 5, 10-718 Olsztyn, Poland; 5 Museum of Natural History, Cascina Vigna, Via S. Francesco di Sales, 188, Carmagnola, Italy; 6 Laboratory of Fish Genetics, Institute of Animal Physiology and Genetics, Czech Academy of Sciences, Rumburská 89, Liběchov, 277 21 Czech Republic; 7 Institut Botànic de Barcelona (IBB-CSIC-ICUB), Passeig del Migdia s/n, 08038 Barcelona, Catalonia Spain; 8 Laboratory of Molecular Epigenetics, Institute of Biophysics, Czech Academy of Science, Kralovopolska 135, 61265 Brno, Czech Republic

**Keywords:** rDNA, Evolution, Chromosome, Fish, *Esox*, Single cell PacBio sequencing

## Abstract

**Background:**

Pikes represent an important genus (*Esox*) harbouring a pre-duplication karyotype (2n = 2x = 50) of economically important salmonid pseudopolyploids. Here, we have characterized the 5S ribosomal RNA genes (rDNA) in *Esox lucius* and its closely related *E. cisalpinus* using cytogenetic, molecular and genomic approaches. Intragenomic homogeneity and copy number estimation was carried out using Illumina reads. The higher-order structure of rDNA arrays was investigated by the analysis of long PacBio reads. Position of loci on chromosomes was determined by FISH. DNA methylation was analysed by methylation-sensitive restriction enzymes.

**Results:**

The 5S rDNA loci occupy exclusively (peri)centromeric regions on 30–38 acrocentric chromosomes in both *E. lucius* and *E. cisalpinus*. The large number of loci is accompanied by extreme amplification of genes (>20,000 copies), which is to the best of our knowledge one of the highest copy number of rRNA genes in animals ever reported. Conserved secondary structures of predicted 5S rRNAs indicate that most of the amplified genes are potentially functional. Only few SNPs were found in genic regions indicating their high homogeneity while intergenic spacers were more heterogeneous and several families were identified. Analysis of 10–30 kb-long molecules sequenced by the PacBio technology (containing about 40% of total 5S rDNA) revealed that the vast majority (96%) of genes are organised in large several kilobase-long blocks. Dispersed genes or short tandems were less common (4%). The adjacent 5S blocks were directly linked, separated by intervening DNA and even inverted. The 5S units differing in the intergenic spacers formed both homogeneous and heterogeneous (mixed) blocks indicating variable degree of homogenisation between the loci. Both *E. lucius* and *E. cisalpinus* 5S rDNA was heavily methylated at CG dinucleotides.

**Conclusions:**

Extreme amplification of 5S rRNA genes in the *Esox* genome occurred in the absence of significant pseudogenisation suggesting its recent origin and/or intensive homogenisation processes. The dense methylation of units indicates that powerful epigenetic mechanisms have evolved in this group of fish to silence amplified genes. We discuss how the higher-order repeat structures impact on homogenisation of 5S rDNA in the genome.

**Electronic supplementary material:**

The online version of this article (doi:10.1186/s12864-017-3774-7) contains supplementary material, which is available to authorized users.

## Background

*Esox*, the only genus in the family Esocidae (Esociformes) contains seven pike species in two monophyletic subgenera (*Esox* and *Kenoza*) with a circumpolar distribution [1]. *Esox americanus*, *E. masquinongy* and *E. niger* live naturally in North America, *E. reicherti* is the only Euroasian esocid endemic to the Amur River basin (Russia and China) while the *E. cisalpinus* (*E. flaviae*) and *E. aquitanicus* are native to Europe [2]. The Northern pike (*E. lucius*) occurs in North America and Eurasia. Its wide distribution and easy access make the Northern pike the most studied esocid species in terms of behaviour, ecology, genetics and evolution. The Northern pike inhabits lakes, rivers and brackish waters. It is an important commercial and recreational species. In the recent years, overexploitation of the natural stocks and climate change has resulted in the dramatic decline of some pike populations [3].

In the Northern pike, nuclear and mitochondrial DNAs exhibit relatively low genetic variability while there is a considerable genetic differentiation among populations [4, 5]. This may be explained by its ecological status–the top predator population size is related to the prey density and/or the bottlenecks that accompanied post-glacial expansion of the pike. Phylogenetic studies confirmed esocids as the closest relatives of the autotetraploid ancestor of salmonid fishes (trout, chars, salmons, ciscoes and grayling) [1, 6]. Both genome size and the number of chromosome arms are about doubled in salmonids when compared to the pike [7, 8]. The Northern pike genome sequence has been recently published [9] and its linkage groups were successfully mapped on the salmonid reference genomes revealing the importance of *Esox* as the pre-duplication outgroup of salmonids [10]. The genus *Esox* possesses 50 acrocentric chromosomes and the number of chromosomal arms (FN) equalled also 50 [11]. The *Esox* karyotype is thus similar to the presumed karyotype of the diploid common ancestor of salmonids. Hypothetically, the salmonid ancestral karyotype after the salmonid-specific whole-genome duplication (WGD) was composed of 100 uni-armed chromosomes (FN = 100). Subsequent diploidization process included vast chromosome rearrangements that have resulted in formation of different karyotypes in the extant salmonids [7, 12]. Centric fusions retaining the number of chromosome arms and decreasing number of chromosome resulted in the formation of Coregoninae and Salmoninae karyotypes. The Thymallinae karyotypes experienced mostly inversions that increased the number of chromosome arms and retained chromosome number close to the presumed ancestral tetraploid karyotype [12]. Therefore, a deep knowledge concerning the *Esox* karyotype and genome organisation is of much importance to reconstruct and understand the complex evolution of salmonid lineages. However, although salmonids are one of the best cytogenetically and molecularly studied fish lineages [13–15], published data on *Esox* sp. chromosomes are limited to basic information concerning chromosome number, their morphology and location of the Nucleolus Organizer Regions (NORs) in *E. lucius*, *E. niger*, *E. masquinongy* and *E. americanus* [7, 11].

The 5S rDNA unit consists of a conserved 120 bp genic region and a more variable intergenic spacer (IGS), also known as Non-Transcribed Spacer (NTS). The genic region contains a tripartite RNA Polymerase III promoter composed of three motifs, Box-A, Internal Element (IE) and Box-C [16], which appear to be conserved throughout the tree of life [17, 18]. Thus the 5S rRNA gene appears to be under extreme selection constrains that maintain structural rRNA features essential for ribosome function and conserved transcription binding sites. The presence of RNA Polymerase III promoter within the short genic region makes the 5S gene a relatively autonomous element prone to change its location on chromosomes. Based on the available data, fish harbour an extraordinary capacity to amplify and spread their rDNA sites across chromosomes - according to the currently assembled animal rDNA database (comprising >900 species of vertebrate, arthropods and molluscs), there are eight fish genera among the top ten species with the largest number of 5S rDNA loci (http://www.animalrdnadatabase.com). In fish, up to 30–40 sites were evidenced in diverged lineages – Cypriniformes, Siluriformes, Characiformes, Salmoniformes, and Esociformes [14, 19–22].

Chromosomal mapping and sequence analysis of 5S rDNA give a powerful tool in genome evolution studies. Two basic models have been proposed for the evolution of rRNA genes (reviewed in [23, 24]). The first model, “concerted evolution”, is based on the observation that rDNA units are uniform within the genome while they differ across the genomes [25, 26]. Under this model, a mutation is rapidly spread across the arrays (genome) or is lost and as a result intragenomic homogeneity of genic and non-coding regions is similar. An alternative, the “birth and death” model has been proposed for multigenic families [27]. According to this model, new genes originate by successive duplications, and are either maintained for a long time or are lost, or else degenerate into pseudogenes. Sequence diversity of coding and spacer regions have been taken as criteria to distinguish between both models and a mixed model of these two has also been proposed [28]. Intragenomic heterogeneity of rDNA paralogs has been correlated with the number of loci in the genome [29]. The known high rDNA mobility across chromosomes [30] has been usually ascribed to various translocation [31] and transposition events [19, 32] as well as to polyploidy and interspecies hybridisation [33]. The latter seems to be the most plausible explanation of the heterogeneity because parental genomes may donate divergent paralogs to their hybrid nucleus. In fish hybrids, the intra- and intergenomic variation was exploited in numerous phylogenetic studies [28, 34].

Despite the wealth of knowledge about 5S rDNA sequence and its chromosome position little is known about the higher-order repeat organisation, in which a block of multiple basic repeat units forms a larger repeat unit of repeats. Here, we exploited PacBio genomic resources now available [9] to determine the higher-order organisation of 5S rRNA genes that to our best knowledge has not been examined to date in any system. We addressed the question of structure, sequence homogeneity and evolution of 5S rRNA genes in two related species *E. lucius* and *E. cisalpinus*. We applied an integrative approach involving classical cytogenetics, molecular biology and *in silico* genomics methods.

## Methods

### Species and sample collections

Following specimens of the Northern pike (*Esox lucius*) were cytogenetically studied: ten young unsexed specimens purchased in a fish farm in Libechov, Czech Republic and 22 8-month old specimens (12 males, nine females and one unsexed) from a fish farm in Olsztyn, Poland. Of the only recently described Southern Pike (*Esox cisalpinus*) we analysed cytogenetically: fourteen young unsexed specimens from the Provincial Fish hatchery of Carmagnola, Italy (young specimens obtained from wild pike parents collected in the Turin Province, determined by G. B. Delmastro and E. Sala), a single specimen from the Po river, Carmagnola, locality Gerbasso, determined by G. B. Delmastro and finally, two specimens determined by G. B. Delmastro that are deposited in the ichthyologic collection of the Muséum National d’Histoire Naturelle (Paris, France).

### Molecular cytogenetics

Molecular cytogenetic methods were carried out independently in laboratories in Poland and Czech Republic. The karyotypes were assessed following standard procedures [14, 35]. Briefly, fish were injected with 0.3% colchicine (Sigma-Aldrich, St. Louis, MO, USA) solution (0.25 ml/100 g body weight) 60 min. before being sacrificed. Portions of cephalic kidneys were removed, placed in 5 ml of a hypotonic solution (0.075 M KCl) and homogenised using glass homogenizers. Cell suspensions were then transferred to the 10 ml glass tubes, hypotonised for 40 min at RT and fixed with freshly prepared ice cold fixative (methanol: acetic acid, 3: 1, v/v). The fixative was changed three times before splashing on microscopic slides. Somatic metaphase plates were prepared by conventional air-drying technique with some modifications [36]. For visualization and description of chromosome morphology, metaphase spreads were stained with 4′, 6-diamidino-2-phenylindole DAPI (Vector, Burlingame, CA, USA).

Based on the number and the quality of the metaphase spreads, we selected two (*E. cisalpinus*) and five (*E. lucius*) individuals for the Fluorescence in situ Hybridisation (FISH) carried out according to [15, 35]. The 5S rDNA probe was obtained by amplification of *Esox* genomic DNA using the forward primer 5S-1: 5′-TACGCCCGATCT CGTCCGATC-3′ and the reverse primer 5S-2: 5′-CAGGCTGGTATGGCCGTAAGC-3′ [37]. For the 28S rDNA probe, following primers were used: 5′-AAACTCTGGTGGAGGTCCGT-3′ and 5′-CTTACCAAAAGTGGCCCACTA-3′. PCR products were purified using the GeneElute PCR Clean-Up Kit (Sigma, USA) and labeled by incorporation of Biotin-16-dUTP (28S) and Digoxigenin-11-dUTP (5S) by nick-translation method (Roche, Switzerland). FISH with 150 ng of rDNA probe per slide was performed with RNase-pretreated and formamide-denaturated chromosome slides. Post-hybridisation wash was performed at 37 °C for 20 min. Detection of FISH signals was performed using Avidin-FITC and anti-Digoxigenin-Rhodamine, Fab fragments, respectively (Roche, Switzerland). Only high quality metaphase cells were examined under Zeiss Axio Imager A1 (Zeiss, Germany) and Nikon 90i (Nikon, Japan) microscopes equipped with epi-fluorescence and digital (Applied Spectral Imaging, Galilee, Israel) and monochromatic ProgRes MFcool (Jenoptic, Germany) cameras, respectively. Images were captured and the electronic processing of the images was performed using the Band View/FISH View software (Applied Spectral Imaging) and Lucia software ver. 2.0 (Laboratory Imaging, Czech Republic). Post-processing elaboration of the pictures was made using CorelDRAW Graphics Suite 11 (Corel Corporation, Canada).

### Cloning and Sanger sequencing

DNA was isolated from blood cells, muscles and fins of adult individuals from several *E. lucius* and *E. cisalpinus* individuals using the classical phenol-chloroform extraction method. The crude DNA fraction was re-purified by DNeasy Blood and Tissue kit (Qiagen, Germany). PCR was used to amplify 5S units from genomic DNA of *E. lucius* and *E. cisalpinus* using PCR as described above. After agarose gel electrophoresis, two fragments of about 220 and 450 bp were visualised in each species. Both fragments were purified and cloned into the pGEM-T vector (Promega, USA). Two clones from each species were sequenced: the short inserts contained monomeric units carrying the 120 bp genic region and an intergenic spacer; the long inserts contained dimmers with two copies of the genic region and a spacer. In addition, one 5S-carrying clone was obtained from the *E. lucius* genomic library prepared by digestion of DNA with the *Ase*I restriction enzyme. The sequences were submitted to the GenBank under the accession numbers (KX950799, KX965715-KX965718).

### Southern and slot blot hybridisation

The procedure followed the protocol described by [38]. The 5S rDNA probe was a 243 bp insert of the 5S_Eci_a clone (GenBank KX965716) from *E. cisalpinus.* The plasmid insert was amplified and labelled with the 32P-dCTP (DekaPrime kit, Fermentas, Lithuania). The probe was hybridised at high stringency conditions (washing 2x SSC, 0.1% SDS followed by 0.1xSSC, 0.1% SDS at 65 °C). The hybridisation signals were visualised by Phosphor imaging (Typhoon 9410, GE Healthcare, PA, USA) and signals were quantified using ImageQuant software (GE Healthcare, PA, USA). The copy number of 5S genes was estimated using slot blot hybridisation. Briefly, the DNA concentration was estimated spectrophotometrically at OD_260nm_ using Nanodrop 3300 Fluorospectrometer (Thermo Fisher Scientific, USA). Concentrations were verified by the electrophoresis in agarose gels using dilutions of lambda DNA as standards. The three dilutions of genomic DNA (100, 50 and 25 ng), together with a serial dilutions of unlabelled plasmid inserts corresponding to the 5S monomers (GenBank KX965715-6), were denatured in 0.4 M NaOH and blotted onto a positively charged Nylon membrane (Hybond XC, GE Healthcare, USA) using a vacuum slot blotter (Schleicher-Schuell, Germany). The probe and the hybridisation conditions and visualisation of signals were the same as described above.

### Intragenomic variation and rDNA copy number determined from NGS reads

The source data was the SRR1197512 archive containing Illumina HiSeq 2000 reads from the whole genome sequencing project of *Esox lucius* (SRX494131, University of Victoria, isolate CL-BC-CA-002) [9]. Sequence downloads and basic read manipulations of the genomic reads were done with the aid of the Galaxy Server [39]. The starting read pool of NGS reads consisted of more than 900 million unpaired reads. Before mapping all reads with Ns, reads less than 90 nt in length or reads below quality Phred scores 30 were removed using ‘QC and Manipulations’ tools at the Galaxy server. The data in FASTQ formats were imported into the CLC Genomics Workbench 6.5.1 (Qiagen, Germany) (CLC). The number of reads was then reduced (Table 1) to decrease computing time using a Sample Reads” command. The high quality reads were mapped to the reference sequences: the 220 bp fragment of 5S rDNA from *E. lucius* (GenBank KX965716) and the 1581 bp fragment of 18S sequence from the pike icefish (*Champsocephalus esox*) (AF518187) using the ‘Map Read Reference’ tool (CLC). The parameter settings were as follows: mismatch cost value: 2, insertion cost value: 3, deletion cost value: 3, with both the length fraction value and the similarity fraction value set at 0.5 and 0.8, respectively. Files with the mapped reads were saved and used for the downstream copy number and SNP analyses. Transcriptomic reads obtained from SRR1228710-12, SRR1228725 and SRR1228729 archives were mapped to 5S genic region (GenBank KX965716) as described above. Relatively few cDNA reads were mapped probably due to the fact that RNA was polyA-filtrated prior to library construction.

**Table 1:** Copy number of rRNA genes in *E. lucius* determined from NGS reads and Southern blot hybridisation

rDNA	Platform^a^/method	Read Archive accession	Total reads	Mapped reads^b^/BLAST hits	GP^c^ (%)	GS^d^ rDNA (Mb)	Copies^e^ (1C)
5S	*IL*	SRR1197513	166,395,976	366,676	0.22	2.42	~20,200
	*PB*	SRR1930096	555,762	29,151	0.16	1.81	~18,000
	*S.blot* ^f^				5	53	~200,000
18S	*IL*	SRR1197513	166,395,976	199,694	0.12	1.32	~820
	*PB*	SRR1930096	555,762	1,436	0.08	0.91	~570

^a^Sequencing platform: *IL* – Illumina; *PB* – PacBio

^b^The number refers to the number of Illumina reads mapped to 5S reference or 5S hits with the PacBio data base

^c^ *GP* – genome proportion

^d^GS – 5S genome space was calculated from GP: genome size (MB) * GP (%), considering 1100 Mb/1C the *E. lucius* genome size

^e^The copies were calculated as follows: GS (bp) divided by size of the 5S unit monomer (220 bp)

^f^Experimental evaluation of copies by the slot blot hybridisation (Additional file 2: Figure S2)

The genome proportion (GP, in percentages) of rDNA was calculated as the number of mapped reads versus total number of reads. The genome space (GS, in megabases) was calculated from the formula: genome size x GP of rDNA units. The copy number was calculated from GS values (in bp) divided by the size of the 5S monomer (220 bp) or part (1581 bp) of the 18S gene. The mapped rDNA units were relatively evenly covered by NGS (each 5S gene nucleotide was covered >3000 x) allowing reliable calculation of genome proportions (not shown).

Variants were called via the ‘Probabilistic Variant Detection’ function tool in CLC using default settings. SNPs were filtered as follows: minimum read coverage–300, count (the number of countable reads supporting the allele)–50, frequency (the ratio of “the number of ‘countable’ reads supporting the allele” to “the number of ‘countable’ reads covering the position of the variant”): ≥5% (high frequency SNPs).

### Analysis of long 5S rDNA arrays within the PacBio reads

We created a BLAST library from the sequence archive SRR1930096 comprising >500,000 PacBio reads. The library was BLASTed against the NGS consensus (the 5S genic region plus the spacer) built from the *E. lucius* Illumina mapped reads. These PacBio reads (2640) were then extracted (primary 5S archive). Because the higher-order organization of units was the primary goal we filtered the primary archive to obtain longer reads of ≥10 kb. This step yielded 286 reads with Phred quality scores Q = 10–11. The average size of read was 12.5 kb; the longest read was 29,914 bp. To determine the number of 5S rRNA genes in each read we used MultiBlast search (e = 1.0) and queried the 286 sequences against the 120 bp 5S NGS consensus (genic region). MultiBlast outputs were exported in the csv format to MS Excel and further processed. Pairwise comparisons of “self to self” read or “read to the 5S rDNA unit NGS consensus” was carried out for each read using the YASS genomic similarity search tool [40]. The alignment parameters were as follows: Scoring matrix (match, transversion, transition, other): +5, −4, −3, −4; gap costs (opening, extension): −16, −4; E-value threshold 0.001. X-drop threshold: 30. In order to reveal degenerate genes, the E-value was increased to 1.0 in some cases. This less stringency search usually resulted in about 15% increase in the number of hits. Outputs were presented as dot-plots.

Spacer variants in long PacBio reads were analysed using the ‘Search Motif Tool’ function in CLC. The search query involved a 10 nucleotide intergenic spacer sequence containing four highly polymorphic positions in the middle. Stringent conditions were applied scoring alignments with more than 90% matches along the 10 nucleotide query. Detected motifs were annotated and counted.

### Phylogenetic and secondary 5S structure analysis

Alignments were built using the 120-bp long genic sequences originating from: (i) clones isolated in this work, (ii) clones from the GenBank, (iii) sequenced rRNA and (iv) cDNA consensus sequences prepared from the mapped transcriptomic reads. ClustalW alignment was implemented within the BioEdit program [41]. A phylogeny NJ tree was constructed using the Seaview program [42]. All calculations were run at default conditions using the Jukes-Kantor model and 1000 replicates.

Phylogenetic NJ trees were constructed from Illumina reads using short 70 bp subregions derived from the 5S genic region (Elu_b clone, position 201–270, GenBank KX965717) and the intergenic spacer (the same clone, position 108–177). The stand-alone BLAST library of SRR1197512 was searched using these subregions as queries. Hit reads were extracted, trimmed for unique lengths (70 bp), sampled to 500 and aligned (‘Multiple Alignment’ tool function of the CLC). Unrooted NJ trees were constructed from aligned reads employing the Jukes-Cantor model and visualised in radial projections. Haplotypic diversity was calculated from aligned reads using the DNASp4 program [43].

Secondary structure modelling was carried out using an online tool at the RNAfold web server ([44], http://rna.tbi.univie.ac.at/). The secondary structures were based on minimum free energy (MFE) calculations using the Turner 2004 model. The program setting was as follows: isolated nucleotides were avoided; vote for dangling energies on both sides of a helix in any case.

### DNA methylation analysis

The purified genomic DNA samples from *E. lucius* (2 individuals) and *E. cisalpinus* (3 individuals) were digested with methylation-sensitive *Hpa*II (sensitive to CG methylation) and its methylation-insensitive *Msp*I isoschizomere (both enzymes are cutting at CCGG). The restriction fragments were hybridised on blots with the alpha[P32]dCTP-labelled 5S probe (Eci_a clone, GenBank KX965716). Control of digestion efficiency was carried out by spiking the *Esox* genomic DNA with a non-methylated plasmid DNA (pBluescript, Stratagen) and subsequent hybridisation with a plasmid probe. Both *Msp*I and *Hpa*II enzymes yielded expected restriction fragments (not shown).

## Results

### Localisation of 5S and 45S rDNA loci and heterochromatin on *Esox* chromosomes

**Fig. 1:**
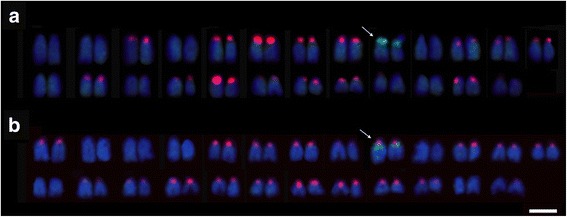
Molecular cytogenetic FISH analysis with rDNA probes to *Esox cisalpinus* (**a**) and *E. lucius* (**b**) chromosomes shown on representative karyotypes. The probes were 5S rDNA in *red* (31 signals in *E. cisalpinus* and 37 signals in *E. lucius*) and 18S rDNA in *green* (two signals in both species marked by *arrows*). Chromosomes are arranged in pairs approximately in decreasing size. More quantitative data on counts of 5S rDNA signals are provided in Additional file 1: Figure S1. Scale equals to 5 μm

Both *E. lucius* and *E. cisalpinus* showed the same number of chromosomes (2n = 50) exhibiting strict acrocentric morphology (FN = 50). We used FISH to determine the number and position of rDNA loci in *Esox* chromosomes using the 5S and 28S rDNA probes (Fig. 1a and b). In *E. cisalpinus*, the 5S rDNA probe hybridised to 30–34 sites (Fig. 1a, quantitative data are summarized in Additional file 1: Figure S1), all in the (peri)centromeric regions. The 28S probe hybridised to (peri)centromeric sites corresponding to NORs on two homologs (single locus) that lacked the 5S rDNA signals (Fig. 1a). In *E. lucius*, the 5S rDNA probe hybridised to 30–38 sites in (peri)centromeric regions (Fig. 1b, quantitative data are summarised in Additional file 1: Figure S1). The 28S rDNA probe hybridised to two NORs that co-localised but not overlapped with 5S signals. The 28S rDNA probe hybridised more distally compared to the 5S rDNA probe. These different patterns of the chromosomal distribution of both major and minor rDNA sequences observed in *E. lucius* and *E. cisalpinus* enabled cytogenetic identification of these two species.

### Massive amplification of 5S gene copies

FISH showed an extraordinary high number of strong 5S probe signals on numerous *Esox* chromosomes indicating a high copy repeat. This observation provoked a question about the 5S gene copy number in both *Esox* genomes. To determine the 5S copy number we first applied computation approach based on the proportion of mapped reads relative to the total reads (Table 1). The 5S rDNA copy number calculated from both data sets (coming from Illumina and PacBio platforms) were in agreement. The 18S rDNA copy number was at least 20 fold lower than that of the 5S rDNA. This is in line with differences in sites number (single 18S site and about thirty six 5S sites, Fig. 1).

We also determined the 5S rDNA copy number using the classical slot blot hybridisation (Additional file 2: Figure S2). Both *E. lucius* DNA isolates yielded more than 200 thousand copies confirming extreme gene amplification. However, the copy number determined by slot blot hybridisation was >10 fold higher than that one calculated from NGS reads.

### Cloning, sequencing and the phylogenetic analysis

**Fig. 2:**
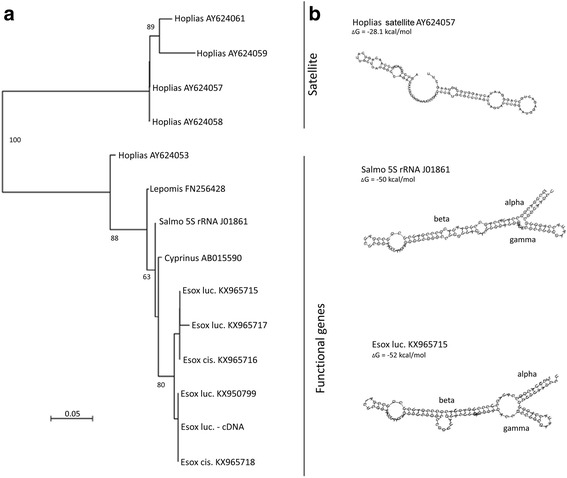
**a** The phylogram constructed from 5S rDNA clones, 5S-related satellites, 5S rRNA and 5S cDNA consensus from the *E. lucius* transcriptome. Bootstrap support of branching (>60%) are indicated. Note, clustering of *Esox* genomic and cDNA clones. Examples of presumed rRNA secondary structures are shown in (**b**) and Additional file 3: Figure S3. Domains of 5S rRNA follow the nomenclature of [17]

A phylogenetic tree (Fig. 2a) was constructed from an alignment comprising several genomic clones from the GenBank, sequenced 5S rRNA genes [37] and a cDNA consensus sequence built from *E. lucius* transcriptomic reads. In addition, a 5S-derived satellite from *Hoplias malabaricus* was included. The sequences grouped into two main clades: (i) The upper clade contained the 5S-derived satellite clones from *H. malabaricus*. Relatively long branches indicate a considerable sequence divergence. (ii) The second clade was formed by a group of genomic clones from different species including those of *Esox*, sequenced 5S rRNA from rainbow trout (*Salmo*) and the cDNA consensus from *E. lucius*. The sequences of clones from *E. lucius* and *E. cisalpinus* were similar both within and across species. Consequently, clones from *E. lucius* and *E. cisalpinus* could not be separated and formed a common branch with the 5S cDNA consensus. Within the functional genes, the *H. malabaricus* sequence (accession AY624053) was relatively well separated from other sequences isolated from *Lepomis*, *Salmo*, *Cyprinus* and *Esox*. This is understandable since *Hoplias* represents the most divergent genus out of the five fish genera analysed [45] indicating that the 5S tree roughly reflected the phylogeny.

### Conserved secondary structures of *Esox* 5S rRNA

Thermodynamic stability of 5S rRNA secondary structure has been considered as an important criterion for gene functionality [46]. In general, the stable three domain structure is an attribute of functional genes. To investigate whether the amplified *Esox* 5S rDNA code for any functional molecules, we predicted the rRNA structures of several *Esox* molecules by computer modelling and results were compared with those of other genera. Indeed, the *E. lucius* clones produced comparable molecule shapes as the 5S rRNA of *Salmo* (rainbow trout) (Fig. 2b) and other potentially functional fish 5S rRNA genes (Additional file 3: Figure S3). The thermodynamic stability was comparable (high) between the species. The 5S-derived satellite from *Hoplias malabaricus* had significantly lower (∆G ~ 28 kcal/mol, in average) thermodynamic stability than the functional genes (typically ∆G ~ 50 kcal/mol) explaining why the satellite structures were vastly different from functional genes of both *Salmo* and *E. lucius*. The typical Y-shape of the fish 5S rRNA was not so pronounced as in other species (Additional file 3: Figure S3) [17, 23]. The structural asymmetry of fish 5S rRNA molecules was apparently caused by a longer beta and shorter gamma domain, respectively.

### Low intragenomic heterogeneity of the 5S genic region contrasts with higher diversity in the intergenic spacers

**Fig. 3:**
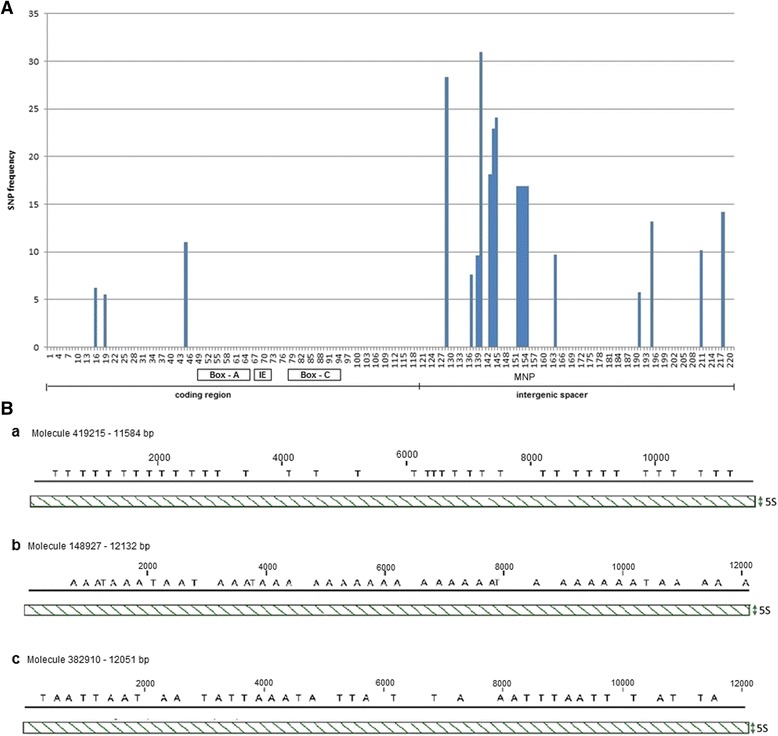
(**A**) Distribution of SNPs along the *E. lucius* 5S rDNA unit. Data were obtained from mutation analysis of Illumina reads (Additional file 4: Table S1). Note, absence of SNPs in the internal controlling region composed of Box-A, IE and Box-C elements. MNP–multinucleotide polymorphism defining two prominent spacer variants (“T” and “A”). (**B**) Distribution of IGS variants in three PacBio reads (a - c). *Slanted lines* indicate tandemly arranged units visualized through the alignment of reads (x-axis) with a 5S gene (y-axis). The position of the “T” and “A”. variants is indicated

In order to determine the intragenomic homogeneity of 5S rDNA we explored the sequenced genome of *E. lucius*. The Illumina reads were mapped to the 5S rDNA reference clone ‘a’ from *E. lucius* (GenBank KX965715) and subjected to the analysis of variance. We considered only high confident SNPs occurring at ≥ 5% frequency, i.e. at least 1000 genes that carry such a variant (considering there may be ~20,000 copies of 5S genes in the genome). Quantification of four kinds of sequence variation (indels and substitutions) along the genic region is shown in Fig. 3A and Table S1 in Additional file 4. Indels were rare and most polymorphisms could be attributed to single nucleotide substitutions. In the 120 bp genic region, only three SNPs, all substitutions, were found. The SNPs located outsides of the regulatory motifs (Boxes A and C and IE) and did not significantly affect secondary structure (not shown). The site containing a T > C mutation at position +45 was also polymorphic among the cDNA reads (Additional file 4: Table S2) suggesting that both variants are expressed. The intragenomic SNPs were approximately five fold more abundant (12.8 SNPs per 100 bp) in the spacer than in the genic region (2.5 SNPs per 100 bp). The 28S rRNA gene was slightly more homogeneous having only 1.2 SNPs per 100 bp of sequence.

In order to determine phylogenetic relationships between the 5S families we constructed haplotypic networks from 500 randomly selected Illumina reads mapping parts of the genic and IGS regions, respectively (Additional file 5: Figure S4A, B). It is evident that the NJ tree constructed from IGS was highly bifurcated compared to the one constructed from genic sequences. The overall diversity expressed as the number of substitutions/100 bp (Pi) was higher (about three-fold) in the spacer region (Pi = 0.0508) than in the genic region (Pi = 0.0145).

### Higher-order organisation of 5S arrays

**Fig. 4:**
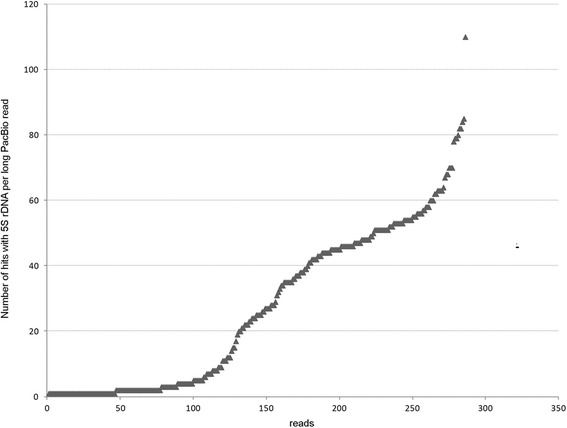
5S rDNA content in long >10 kb PacBio reads of *E. lucius*. The total number of reads was 286. The sequences were queried with the 5S consensus (genic part). The MultiBlast search yielded 7768 alignments (about 43% of total 18,000 hits, Table 1) here considered as gene copies

**Fig. 5:**
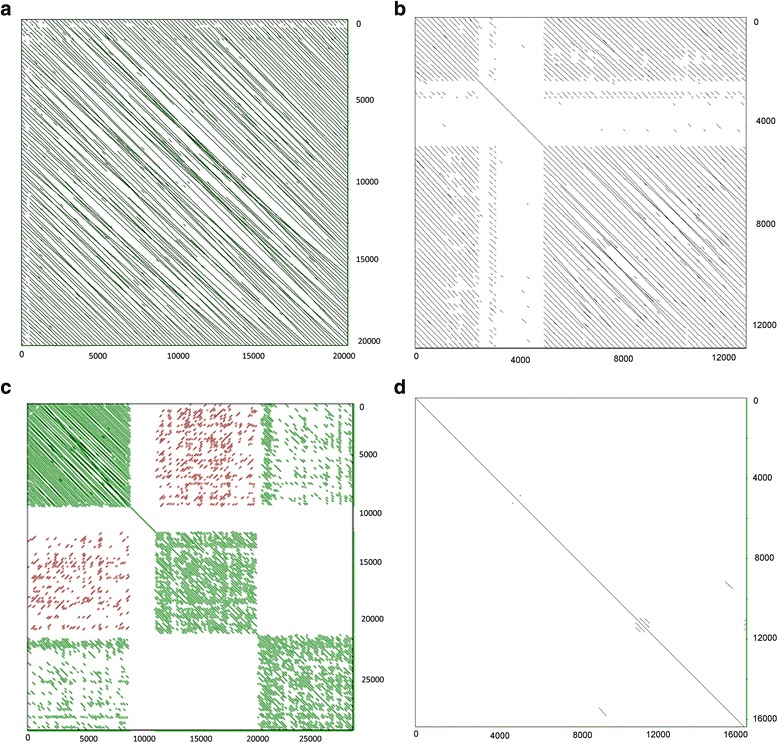
Higher-order organization of 5S rDNA arrays in *E. lucius*. Self-to-self comparison of long PacBio molecules representing four groups: **a** Group I molecule #531194 (19,172 bp) containing the longest uninterrupted block of 5S genes (87). **b** Group II molecule #421211 (13,043 bp) containing two blocks of tandem repeats (52 genes) separated by a ~2 kb spacer. **c** Group III molecule #496426 (29,914 bp). It represents the longest PacBio read available harboring 110 5S copies; **d** Group IV molecule # 49823 (16,372 bp) containing large part of unique sequence plus five copies (three in tandem) of 5S rDNA. Coordinates are in base pairs

To date, the regularity of 5S tandems and distribution of variants of arrays in the genome have not been assessed due to technical difficulties related with sequencing of long and repetitive molecules. Only now, the single molecule sequencing technologies, such as the PacBio, appear to be suitable for such studies since they generate longer reads than other sequencing platforms. We took advantage of the availability of the PacBio-sequenced *E. lucius* genome (ENA archive SRR1930096) and addressed the question of distribution of 5S intergenic spacer variants (i) and higher-order organisation of 5S arrays (ii):

i. Previous SNP analysis revealed variation in the intergenic spacer region (Fig. 3A). At the position 152–155, the tetranucleotide TCCT > AGGA variation corresponded to a more abundant (83%) “T” (TCCT motif) and the less (17%) abundant “A” (AGGA motif) variants (Additional file 4: Table S1). We determined the distribution of these variants in three randomly selected PacBio molecules that showed relatively high quality scores (*Q* = 11) and hence may be used for analysis of variants (Fig. 3B). All three molecules (a, b, c) had a comparable number of tandemly arranged genes ranging 50 to 55. The molecule in (a) contained 50 complete units out of which 37 had the “T” spacer variant. Thirteen units had neither of the two variants probably due to mutations or sequencing errors. The molecule in (b) had 51 complete units. Out of these, 33 units had the “A”, three units had the “T” and 15 had other variants. The molecule in (c) was the most heterogeneous comprising 55 units out of which 20 can be assigned to the “T” and 19 to the “A” variants. Thus, variant composition and homogeneity differ from array to array.
ii. The higher-order organisation of 5S repeats was investigated in 286 PacBio molecules extracted from the 5S rDNA BLAST search dataset and size-filtrated for 10–30 kb. This subset representing about 43% of total *E. lucius* 5S rDNA was analysed for gene richness (Fig. 4). The 5S genes number varied markedly (1–110) between individual reads indicating differences in genomic organisation of repeats. To analyse the higher-order repeat organisation, each sequence was subjected to self to self comparison (Additional file 6: Figure S5). Based on the resulting dot-plot profiles, four types of higher-order organisation can be distinguished (Table 2 and Additional file 7: Tables S3): (i) Group I, molecules containing continuous blocks of 5S tandem repeats spanning the entire read length (Fig. 5a). (ii) Group II, molecules containing one or several blocks of 5S head to tail tandems plus variable portions of unrelated mostly unique sequences (Fig. 5b). In one read, the 5S block was attached to another block of 5S-unrelated tandems (Additional file 7: Table S3). (iii) Group III, molecules harbouring invertedly repeated blocks of 5S tandems that may or may not be separated by spacers (Fig. 5c and Additional file 8: Figure S6). (iv) Group IV, containing no longer blocks (Fig. 5d), but rather dispersed 5S copies. Despite relative abundance (37% reads) the 5S gene richness was low (~4%) in this group (Table 2). In contrast, gene representation in reads bearing long blocks (Groups I–III) was high (96%). Pairwise comparisons of reads with the 5S reference (the genic region consensus built from Illumina reads) allowed us to determine variation in length of intergenic spacers. The three major spacer length variants were identified: (i) Short 95–116 bp variant occurring in 7035 (94%) units; (ii) Long 321–340 bp variant found in 357 (5%) units; (iii) Ultralong 1153–1209 bp variant found in 65 (1%) units (Additional file 7: Table S3). The sequences of short and long spacer variants were unrelated. Except for one read, spacer variants formed independent arrays.

**Table 2:** Classification of PacBio reads according to 5S rDNA higher-order organisation

Group	5S arrangement in a read^a^	Number of reads (percentage)	Number of 5S genes (percentage) ^b^
I	Tandem repeats, no unique DNA	95 (33)	4539 (58)
II	Tandem repeats + unique DNA	72 (25)	2352 (30)
III	Blocks of inverted repeats	11 (4)	639 (8)
IV	Dispersed or short (<5 units) tandems	108 (38)	274 (4)
Total		286 (100)	7769 (100)

^a^The data sets and read annotations are in Additional file 7: Table S3

^b^The number of genes in reads was determined based on MultiBlast search using the 5S genic region (NGS consensus) as a query

### The majority of 5S rRNA genes are highly methylated

**Fig. 6:**
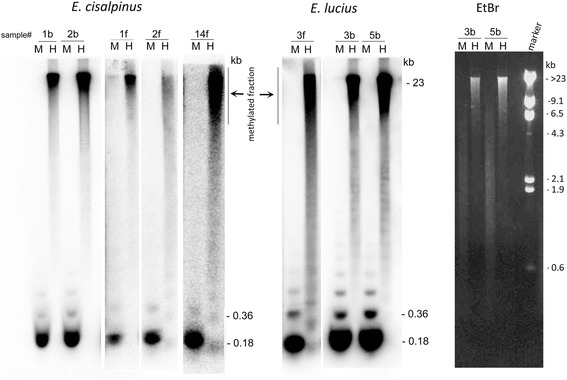
Methylation analysis of 5S rRNA genes by the methylation-sensitive *Hpa*II (H) restriction enzyme and its methylation-insensitive *Msp*I (M) isoschizomere. The *left two panels* show Southern blot hybridisation with the 5S rDNA probe; the *right panel* (EtBr) shows ethidium bromide-stained DNA fragments before the hybridisation. The probe hybridised with a ~200 bp *Msp*I fragment corresponding to a monomer. Hybridisation signals with the *Hpa*II fragments were of high molecular weight. Samples “f” and “b” were fin and blood isolates, respectively. The samples from fin tissue were slightly degraded which probably accounted for smear signals

The methylation status of 5S rDNA genes in *E. lucius* and *E. cisalpinus* was determined by enzymatic digestion of genomic DNA with methylation-sensitive *Hpa*II and insensitive *Msp*I. In all samples, the probe hybridised to high molecular weight bands after the digestion of DNA with methylation-sensitive *Hpa*II (Fig. 6). In contrast, a major low molecular weight band of about 220 bp was visible after the digestion with *Msp*I. The slow-migrating oligomeric *Msp*I bands were faint confirming a high homogeneity of arrays. The near complete resistance of 5S rDNA to *Hpa*II digestion indicated high level of methylation of most of the units. There were no differences in methylation profiles between the blood and fin tissues. The globally high methylation level is evident from ethidium-bromide stained DNA fragments. Most *Hpa*II-fragments migrated as high molecular weight relic while the DNA was relatively efficiently digested into shorter fragments with methylation-insensitive *Msp*I.

## Discussion

### Massive amplification of 5S rRNA genes in *Esox lucius*

In animals, the number of rRNA genes typically reaches up to hundreds of copies [46–48]. It is therefore striking that these *Esox* species harbour tens of thousands of 5S rDNA copies. The actual copy number could be even higher since the experimental copy number estimates exceed 200 thousand copies forming about 5% of the *Esox* genomes. The apparent discrepancy (more than 10 fold) between NGS and slot blot hybridisation could be attributed to the fact that tandem repeats are typically underrepresented in the DNA sequencing libraries [49, 50]. The second explanation could be potential variation in 5S copy number in different populations of *E. lucius*. In our study, the copy number was calculated from the whole genome sequencing dataset of an American population while the experimental copy number estimation was performed in different individuals of European origin. Thus, we cannot exclude inter-population differences in copy numbers, already reported in mammals [51]. In any case, the number of 5S rRNA genes exceeding tens of thousands of copies is far more than seen in most metazoans (http://www.animalrDNAdatabase.com).

The 5S rDNA copy number, sequence and position on chromosomes are similar in *E. lucius* and *E. cisalpinus*. The genomic spreading of 5S rDNA was not accompanied by any concomitant expansion of 45S rDNA whose copy number was limited to about 800 (Table 1). Independent amplification of both types of rDNA has also been reported in other fish genera [19, 21] suggesting that concerted amplification of 45S and 5S rDNA [51] may not be operating in fish or it is limited to particular groups. Related salmonid fishes harbour far more 45S than 5S loci [13–15, 36]. Thus, it is reasonable to suggest that amplification of 5S must have occurred after the divergence of a common *Esox* ancestor from the rest of salmonids.

### Possible epigenetic regulation of amplified 5S rRNA genes by DNA methylation

The extremely high copy number of 5S genes in *Esox* resembles that of some amphibians [52]. However, in amphibians amplified repeats are attributed to be pseudogenes while most repeats we analysed here are probably capable to encode functional 5S rRNA. This was evidenced by their high intragenomic homogeneity, good matches between genomic and transcriptomic reads and conserved thermodynamically stable secondary structures. This raises an important question about their transcription regulation. In mammals, only about one hundred of 45S rRNA genes are estimated to be transcribed at any one time (for review see [53]). In *Danio rerio* (zebrafish) only twelve 5S copies appear to be active in oocytes while a large number of genes in another locus are silenced and activated only at later developmental stages [54]. Perhaps, transcription activity of amplified genes in *Esox* might be regulated via epigenetic mechanisms as well. Indeed, 5S genes in both *E. lucius* and *E. cisalpinus* were heavily methylated at CG motifs suggesting that a powerful epigenetic system has evolved in this genus. There were no apparent differences in methylation levels between different tissues reported in other systems [55] suggesting that most genes are evenly methylated. However, our restriction enzyme-based methylation assay reveals global level of 5S rDNA methylation while its resolution is unable to detect changes at the single gene level. It is worth noting that sequence polymorphisms are located within the first half of the 5S gene both in *E. lucius* (Fig. 3) and *D. rerio* [54]. Therefore, developmental regulation of 5S rDNA expression cannot be excluded in *Esox*.

### The 5S genes are organised in large blocks of variable sequence homogeneity

Tandem arrays of repeated sequences are generally considered as problematic regions often refractory to in depth genomic analysis. Hence, the organisation of repeat variants in the genome has been intensively discussed [47, 56, 57]. Based on conventional Southern hybridisation methods, repeat variants are believed to form separate arrays [58]. However, at the genomic scale evidence is missing due to technical difficulties related to sequencing of long and repetitive molecules. In *E. lucius*, variation in the 5S intergenic spacer was about 5-fold higher than in the genic region indicating relaxed selection constraints on intergenic spacers. We took the single cell PacBio sequencing approach to study distribution of spacer variants in tandem arrays in this species. Being aware that the randomly occurring sequencing errors are relatively high (~13% in DNA polymerase single pass) using this technology [59] making sequence polymorphisms difficult to interpret. However, sequencing errors cannot account for all the variation observed in our data sets. This is because SNPs (spacer variants) residing in the array were regularly phased (Fig. 3B) while sequencing errors are distributed randomly [59]. Secondly, the type and position of multinucleotide variation detected in long PacBio molecules were similar to those detected in high quality Illumina reads. Thus, the analysis of PacBio reads seems to be an adequate way to address the question of distribution of major variants. We found that 5S spacer variants form both separate and mixed arrays. Strict tandem arrangement of 5S units in the heterogeneous arrays suggests that single or multiple nucleotide polymorphisms do not impair array regularity. In contrast, major spacer length variants tend to form independent arrays (Additional file 7: Table S3). Interestingly, in *Arabidopsis* where 5S loci are located on three chromosomes, a similar block-like structures composed of homo- and heterogenous arrays were detected in BAC libraries [60] and recent PacBio sequencing revealed substantial spacer polymorphisms in the 45S rDNA intergenic spacers located on two chromosomes [61]. These results suggest that the higher-order repeat structure may be evolutionary conserved. Despite the variation, evolution of 5S rRNA genes in *Esox* almost certainly fits the concept of concerted evolution considering that tens of thousands of copies are present in the genome and the SNPs being relatively infrequent. Relative heterogeneity of some arrays can be explained by reduced efficiency of interlocus recombination and/or less stringent selection constrains imposed on the spacers.

### Mechanisms of arrays spreading

Accumulation of repeats at similar chromosomal positions raises questions on the mechanisms mediating spreading and homogenisation of 5S rDNA units. Several hypotheses can be drawn:

i. Chromosome location may affect recombination rate and homogenisation of 5S arrays. In mouse, a huge copy number variability in 45S rRNA genes has been associated with their purely centromeric location in mostly acrocentric/telocentric chromosomes [62]. Thus, it has been proposed that DNA breaks may appear quite frequently in such located rDNA sequences leading to translocations of rDNA to other chromosomes. In *Esox*, 5S loci are uniformly located on short arms of nearly all acrocentric chromosomes. By analogy, in *Esox*, interlocus recombination of 5S genes could be driven by their (peri)centromeric position in acrocentric chromosomes.
ii. 5S rRNA genes are able to multiply and integrate into other areas of the genome using a mechanism similar to retrotransposition among others. The 5S rRNA genes and SINEs harbor the same type of internal RNA polymerase III promoter [63]. On the top of that, some authors [64, 65] found a unique class of SINEs that have been formed by fusion of a 5S rRNA gene and a LINE, showing that 5S and retroelements may interact. In support, SINE elements with 5S features were reported in several fish [54, 64] and a retroelement was co-localised with 5S loci on *Erythrinus erythrinus* (red wolf fish) chromosomes [19] and recently among members of another fish genus, *Gymnotus* [66]. In the future it will be interesting to analyse inter-block spacers in Group II reads (those containing one or few blocks of 5S genes head to tail, plus unrelated sequences) for the presence of transposable elements which may support their potential role in 5S rDNA mobilisation.
iii. 5S genes spread through extrachromosomal replication and reintegration in new locations. Covalent extrachromosomal circles of rDNA have been identified in diverged biological taxa [67, 68]. In *Xenopus laevis* rDNA is known to replicate extrachromosomally during development [69]. In our datasets some loci (4% PacBio reads) contained invertedly repeated large blocks of 5S tandems. There is experimental evidence that extrachromosomal DNA can be generated by replication errors at the inverted repeats [70]. Therefore, one can hypothesize that the homogenisation of 5S loci across *Esox* chromosomes is mediated by the initial replication block at the inverted repeat, excision, extrachromosomal replication and reintegration by homologous or non-homologous recombination into a new genomic location. Large palindromes may thus transpose and seed 5S blocks to distal locations. Supporting this, a recent study of a human centromeric satellite (using long read PacBio sequencing) showed increased frequencies of inversions in acrocentric chromosomes compared to other chromosomes [71]. Perhaps, acrocentromeric positions of rDNA could be particularly vulnerable to such rearrangements and/or inversions. This model may also explain why interlocus homogenisation of rDNA often occurs without extensive chromosome rearrangements [30, 72].

### Long term scenario of arrays evolution

i. It can be envisaged that as long as 5S rRNA genes undergo interlocus homogenisation the number of loci would remain relatively constant. This is likely happening in both closely related species *E. lucius* and *E. cisalpinus* that show similar number of genes, sequence of the loci (including intergenic spacers) and their chromosome positions. Yet, at the cytogenetic level, small differences were noted: in contrast to *E. lucius*, *E. cisalpinus* lacked the co-localisation of 5S and 45S rDNA. Given that the 45S loci occur on homeologous chromosomes it follows that there was either 5S locus gain (*E. lucius*) or locus loss (*E. cisalpinus*) after the separation from the common ancestor. Perhaps, adjacent blocks to 45S and 5S could be represented by an unstable chromatin configuration leading to breaks and chromosomal translocation.
ii. As methylated cytosines are more susceptible to mutations [73] we may expect gradual accumulation of C > T and G > A mutations in the *Esox* 5S genes and their subsequent pseudogenisation. Indeed, in some plant species most rRNA genes were converted into pseudogenes [74]. However, we have no evidence for such significant erosion in *Esox* 5S rDNA despite its high methylation (methylation-induced mutations were not significantly enriched in *Esox* 5S rDNA, *p* > 0.05). Moreover, two major variants of a genic region seem to be expressed suggesting that not all mutations automatically lead to pseudogenisation. It is likely that *Esox* genomes seem to be in a dynamic phase of evolution, where most pseudogenes are being removed by genetic recombination.
iii. Amplified 5S genes acquire centromeric function. Retrotransposons and RNA-polymerase-III-transcribed genes, including tRNA and 5S rRNA (the so called Pol III genes) have been found to be associated with centromeres in fission yeasts. Furthermore, results of some studies suggest a functional link between the centromeric localisation of the Pol III genes and chromosome condensation resulting in the proper assembly of mitotic chromosomes [75]. In the fish *H. malabaricus*, 5S genes gave rise to an independent satellite with apparently centromeric function [76]. Similar conversion might have happened in other species [77, 78] as well. Thus, if 5S rDNA is supportive to the centromeric function and plays a role in the assembly of mitotic chromosomes then its “invasions” to centromeric positions might be favored in evolution. Natural selection would select those variants that bind centromeric histones and adopt centromere-specific chromatin configuration. Relative heterogeneity of some blocks bearing degenerated units (Fig. 5c and Additional file 6: Figure S5) suggests that the process of satellite formation could have already been started. It is also possible that satellite repeats may arise from orphanised degenerated 5S insertions accounting for about 4% of *E. lucius* rDNA (Table 2).

## Conclusions

In two European *Esox* species we have witnessed the extreme amplification of 5S rRNA genes reaching up to tens of thousands of copies and their distribution across more than half of the chromosomes. Such a high number is exceptional in animals, generally thought to contain moderate number of these genes. Most of the amplified genes appear to be functional and heavily epigenetically modified. Detailed analysis of long PacBio reads suggests a considerable variation in the phasing of unit variants and in the arrangement of large blocks of 5S tandem repeats. These higher-order structure polymorphisms may potentially influence the expression and homogenisation of these genes.

## Additional files

Additional file 1: Figure S1. Summary on counts of FISH signals of 5S rDNA on chromosomes of two individuals of *E. cisalpinus* (Eci1 and Eci2) and five individuals of *E. lucius* two of which originated from the Czech Republic (EluCz3, EluCz5) and three from Poland (EluP8, EluP9, EluP15). (PDF 309 kb)

Additional file 2: Figure S2. Estimation of 5S rDNA copy number by slot blot hybridisation. The results are shown for two independent genomic DNA isolates and two 5S insert standards (*E. lucius* and *E. cisalpinus*). The 5S rDNAs (genic + spacer regions) account for about 5% of *E. lucius* genome equaling to about 250,000 copies (the data collected from two blot replicates and averaged). (PDF 267 kb)

Additional file 3: Figure S3. Secondary structure models of 5S rDNA molecule for fish (A) and non-fish species (B). (PDF 186 kb)

Additional file 4: Tables S1 and S2. Type, position, frequency and coverage of 5S rDNA polymorphisms. Table S1 – Analysis of genomic Illumina reads. Table S2 – Analysis of transcriptomic Illumina reads. SNPs along whole 5S units (genic and the intergenic spacer) were analyzed using genomic reads; for the transcriptome, only the genic region was considered. Note, high number of SNPs in spacer region compared to the genic region despite the shorter length (Table S1). SNP – single nucleotide polymorphism; MNP – multiple nucleotide polymorphism. (PDF 870 kb)

Additional file 5: Figure S4. Phylogenetic NJ trees constructed from 500 aligned Illumina reads derived from the 5S genic part (A) and an intergenic spacer (B). Well supported branches (bootstrap >60%) are indicated by blue arrows. (C) A sequence of the 5S clone b from *E. lucius* (GenBank KX965717.1) with highlighted subregions used in the phylogenetic analysis. (PDF 856 kb)

Additional file 6: Figure S5. Dot plot diagrams resulting from self to self comparison of long PaBio reads. (PDF 1371 kb)

Additional file 7: Table S3. Analysis of higher-order repeat structure of 5S rDNA using long (≥10 kb) PacBio reads. The selected sequences are ordered according to lengths (descending). Number of gene copies in reads was determined by MultiBlast. Arrangement was assessed by visual inspection of dot plot matrices (Additional file 6: Figure S5). Grouping followed the nomenclature in Table 2. Intergenic spacer variants: S–short (95–116 bp); L–long (321–340 bp) and UL–ultralong (1153–1209 bp). (PDF 471 kb)

Additional file 8: Figure S6. A group III molecule #499258 (19,900 bp) organised in two large immediately linked inverted blocks of tandem repeats. Green and red slanted lines indicate direct and inverted orientation of units, respectively. (B) The junction region alignment to 5S. Note, absence of any 5S-unrelated sequence between the inverted repeats. Note, a partial deletion of IGS in the third copy. (C) Nucleotide sequence of the junction region with annotated genic sequence (brown) and IGS (green). (PDF 291 kb)

## Abbreviations

∆G: Change in Gibbs free energy
FISH: Fluorescence In situ Hybridisation
FN: Fundamental number, number of chromosomal arms
GB: Gigabase
GP: Genome proportion
GS: Genome space
IE: Internal Element
IGS: Intergenic spacer
IL: Ilumina sequencing
MB: Megabase
MNP: Multiple nucleotide polymorphism
NGS: Next Generation Sequencing
NJ: Neighbour Joining
NOR: Nucleolus Organizer Region
NTS: Non-Transcribed Spacer
PB: PacBio sequencing
rDNA: Ribosomal RNA genes
SNP: Single nucleotide polymorphism
WGS: Whole Genome Duplication
