# Supplementary material for: Higher-order organisation of extremely amplified, potentially functional and massively methylated 5S rDNA in European pikes (Esox sp.)
Source: BMC Genomics. 2017 May 18;18:391. doi: 10.1186/s12864-017-3774-7 (PMC5437419; doi:10.1186/s12864-017-3774-7)

Figure S1. Summary on counts of FISH signals of 5S rDNA on chromosomes of two individuals of *E. cisalpinus* (Eci1 and Eci2) and five individuals of *E. lucius* two of which originated from the Czech Republic (EluCz3, EluCz5) and three from Poland (EluP8, EluP9, EluP15).

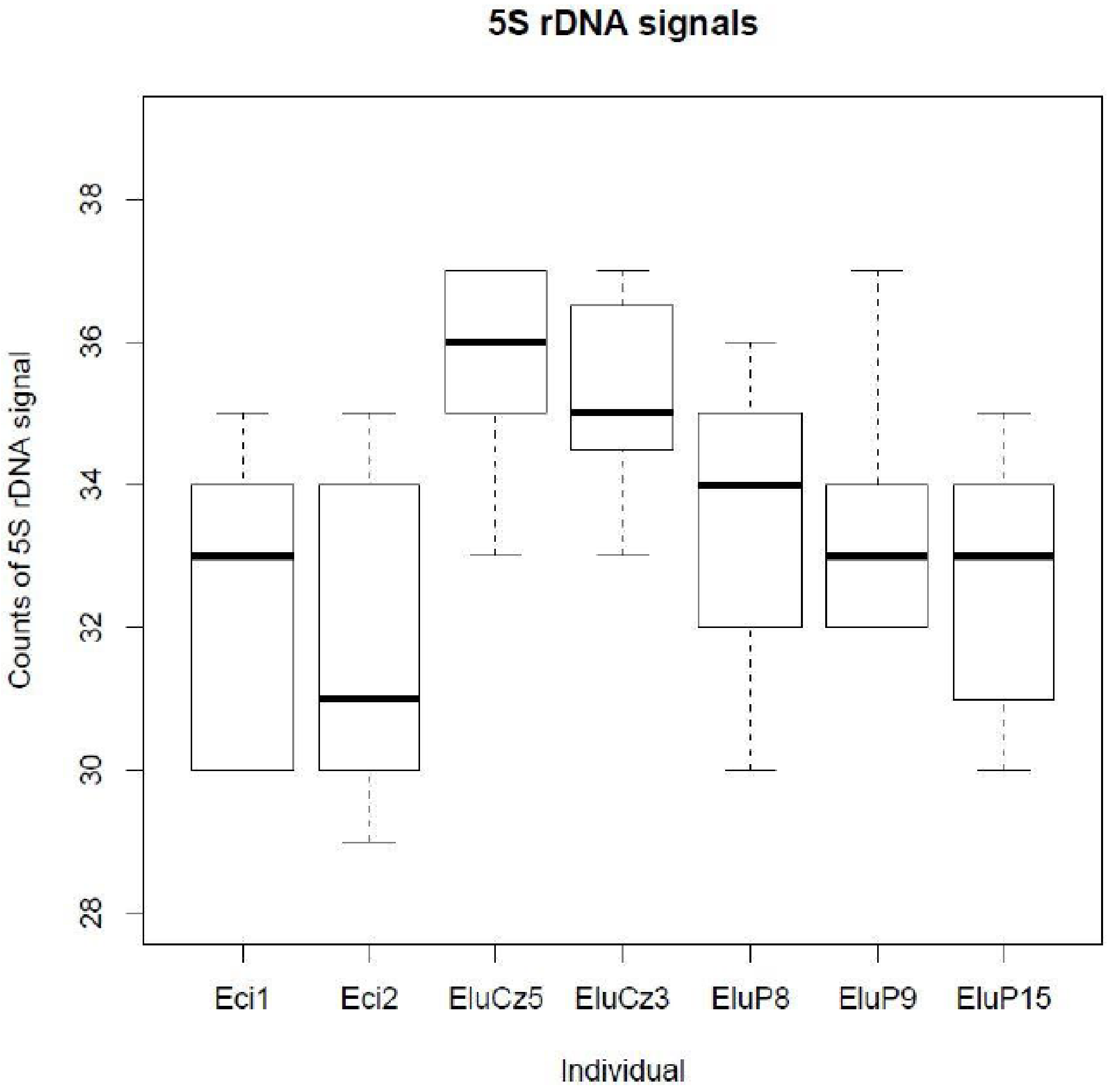

Supplement: Supplementary file 1 — Summary on counts of FISH signals of 5S rDNA on chromosomes of two individuals of E. cisalpinus (Eci1 and Eci2) and five individuals of E. lucius two of which originated from the Czech Republic (EluCz3, EluCz5) and three from Poland (EluP8, EluP9, EluP15). (PDF 309 kb) [file 12864_2017_3774_MOESM1_ESM.pdf]
