# Supplementary material for: Higher-order organisation of extremely amplified, potentially functional and massively methylated 5S rDNA in European pikes (Esox sp.)
Source: BMC Genomics. 2017 May 18;18:391. doi: 10.1186/s12864-017-3774-7 (PMC5437419; doi:10.1186/s12864-017-3774-7)

Figure S2. Estimation of 5S rDNA copy number by slot blot hybridisation. The results are shown for two independent genomic DNA isolates of *E. lucius* from the Czech populalion and two 5S insert standards (*E. lucius* and *E. cisalpinus*). The 5S rDNAs (genic + spacer regions) account for about 5% of *E. lucius* genome equaling to about 250,000 copies (the data collected from two blot replicates and averaged).

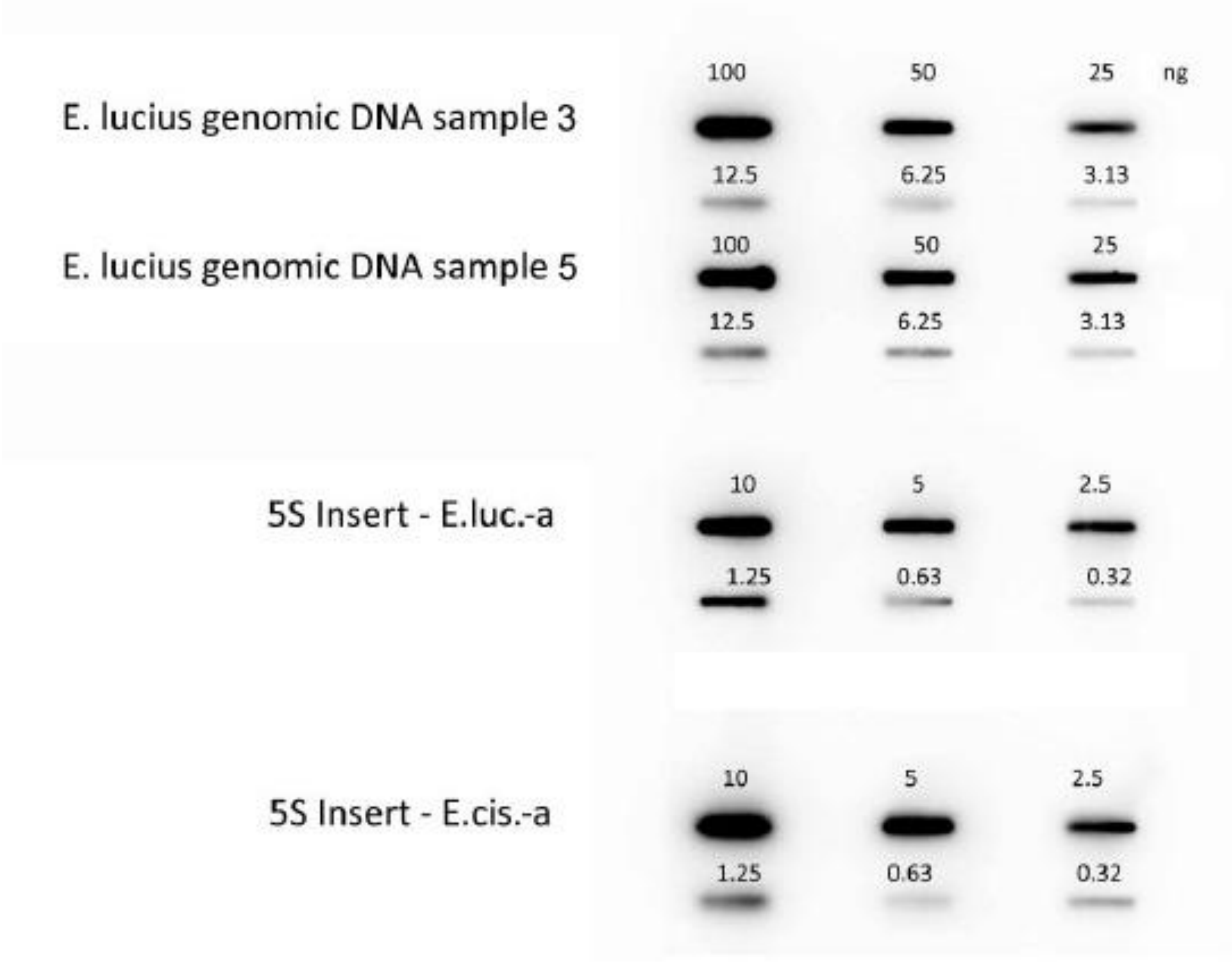

Supplement: Supplementary file 2 — Estimation of 5S rDNA copy number by slot blot hybridisation. The results are shown for two independent genomic DNA isolates and two 5S insert standards (E. lucius and E. cisalpinus). The 5S rDNAs (genic + spacer regions) account for about 5% of E. lucius genome equaling to about 250,000 copies (the data collected from two blot replicates and averaged). (PDF 267 kb) [file 12864_2017_3774_MOESM2_ESM.pdf]
