# Supplementary figures and images for: Higher-order organisation of extremely amplified, potentially functional and massively methylated 5S rDNA in European pikes (Esox sp.)
Source: BMC Genomics. 2017 May 18;18:391. doi: 10.1186/s12864-017-3774-7 (PMC5437419; doi:10.1186/s12864-017-3774-7)

**Figure S3.** Secondary structure models of 5S rDNA molecule for fish (A) and non-fish species (B)

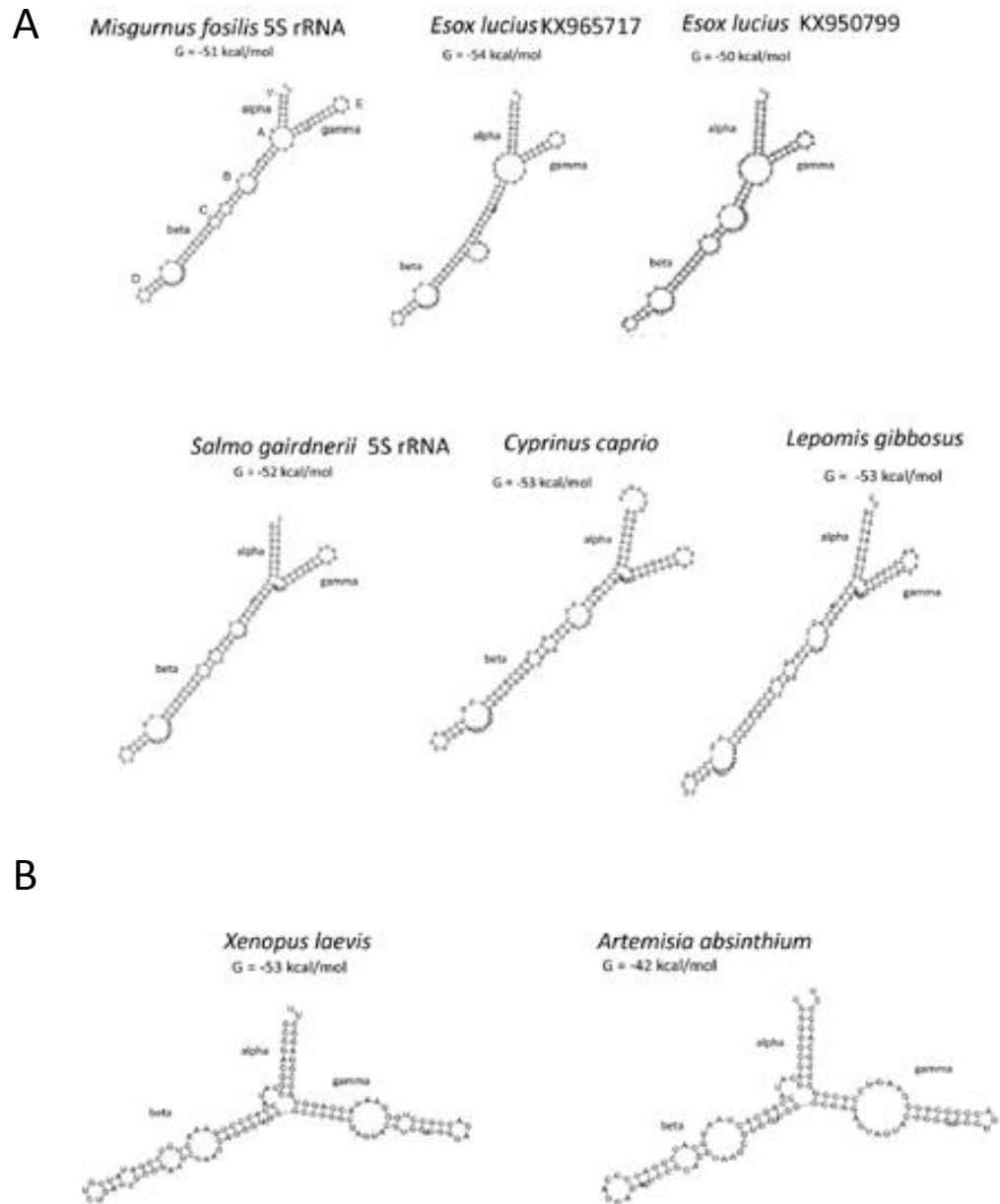

Supplement: Supplementary file 3 — Secondary structure models of 5S rDNA molecule for fish (A) and non-fish species (B). (PDF 186 kb) [file 12864_2017_3774_MOESM3_ESM.pdf]

**Figure S5.** Dot plot diagrams resulting from self to self comparison of long PacBio reads.

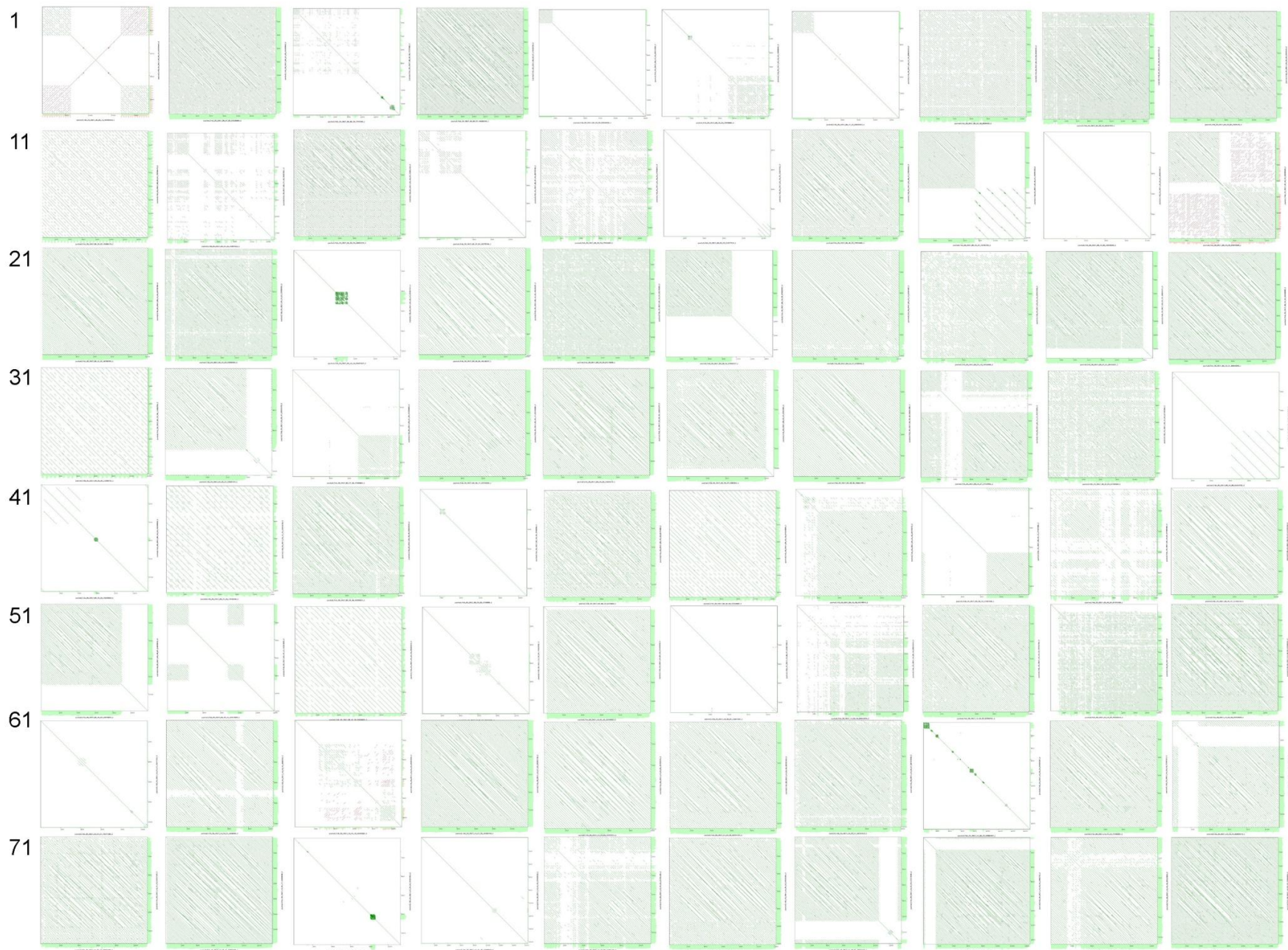

91

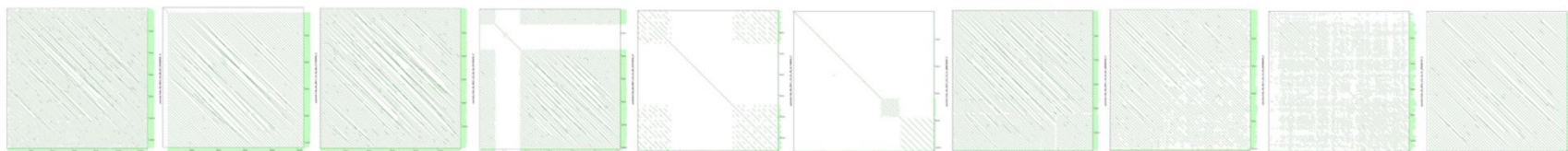

101

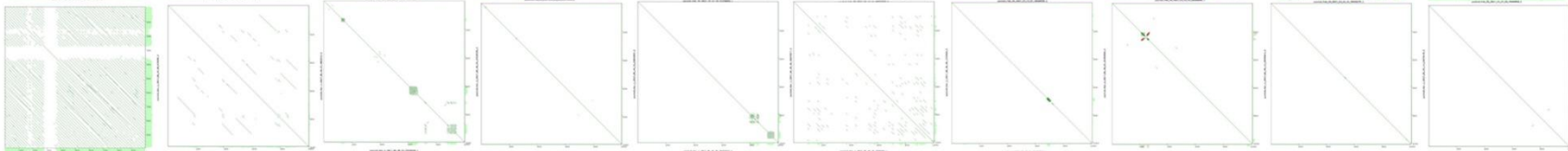

111

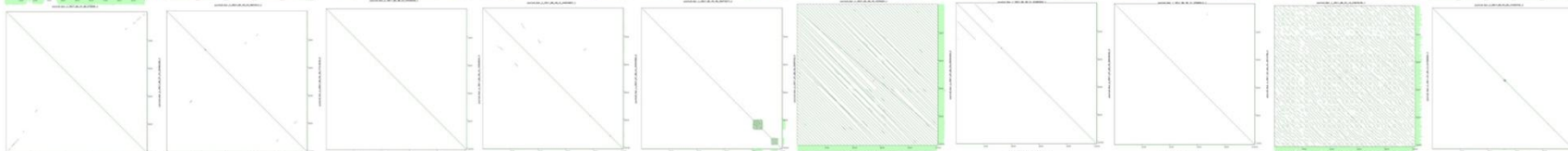

121

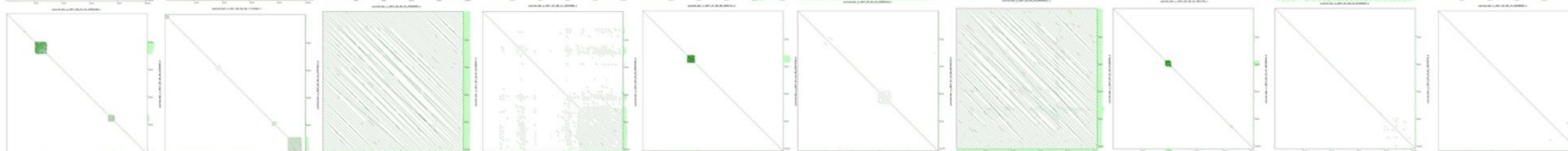

131

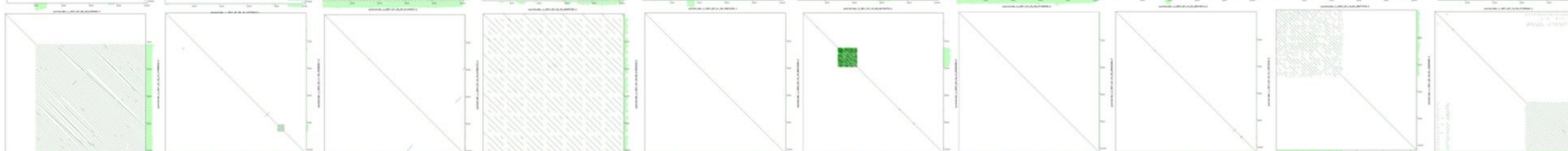

141

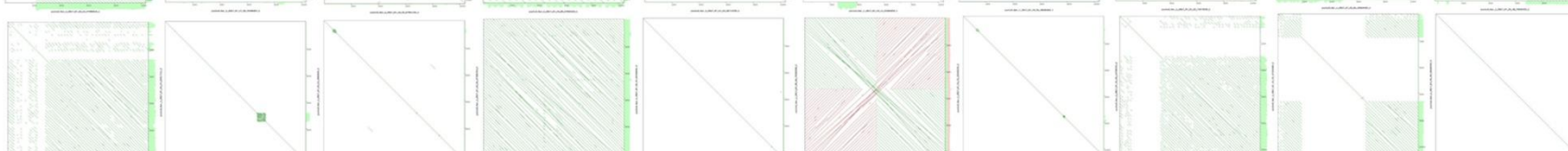

151

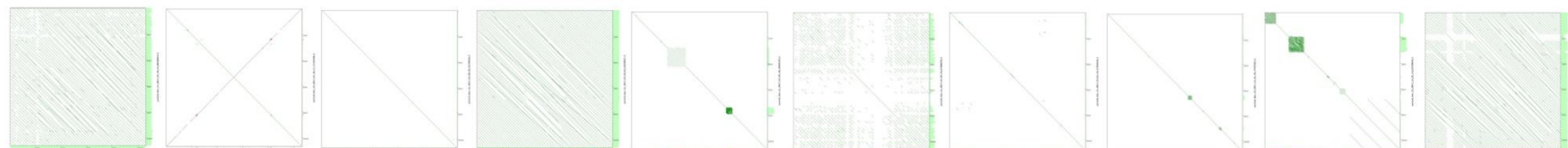

161

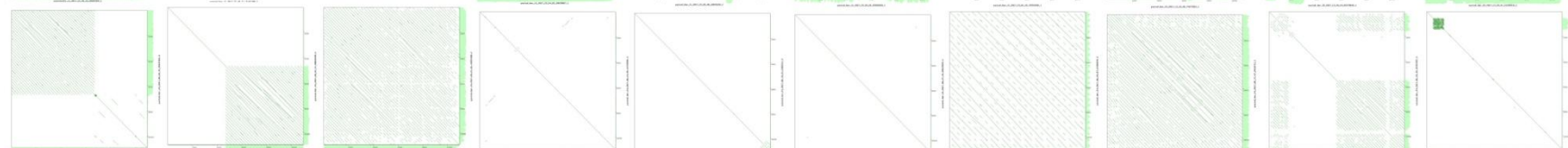

171

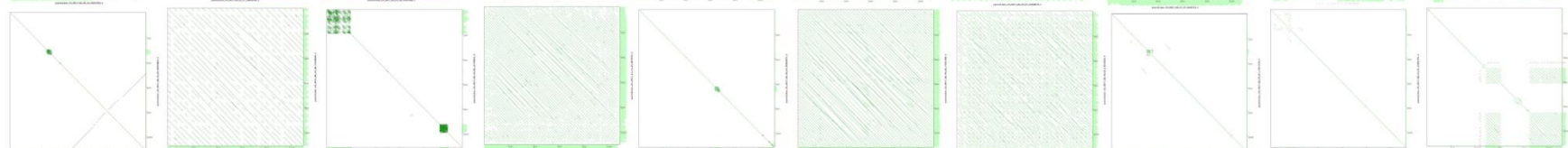

181

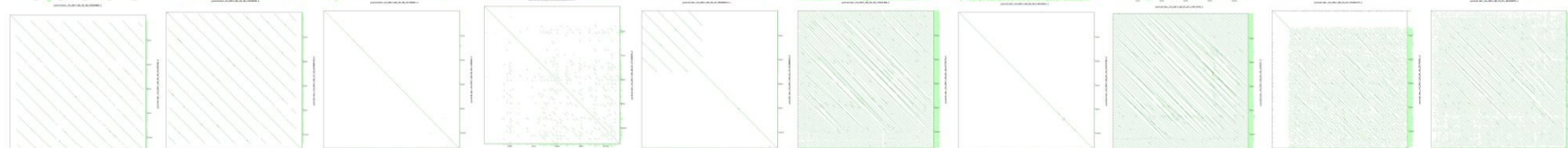

191

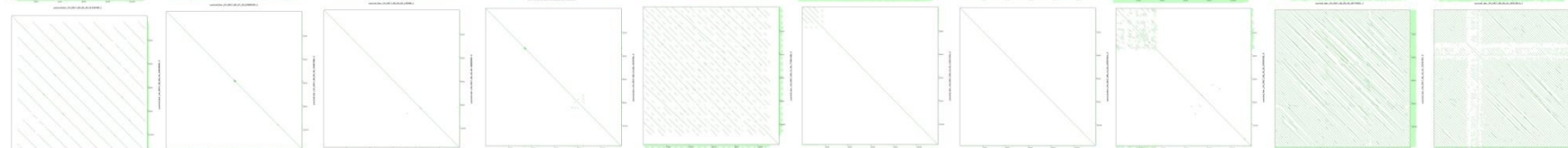

201

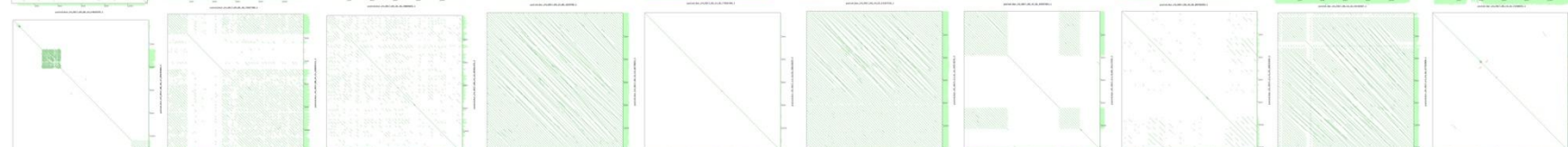

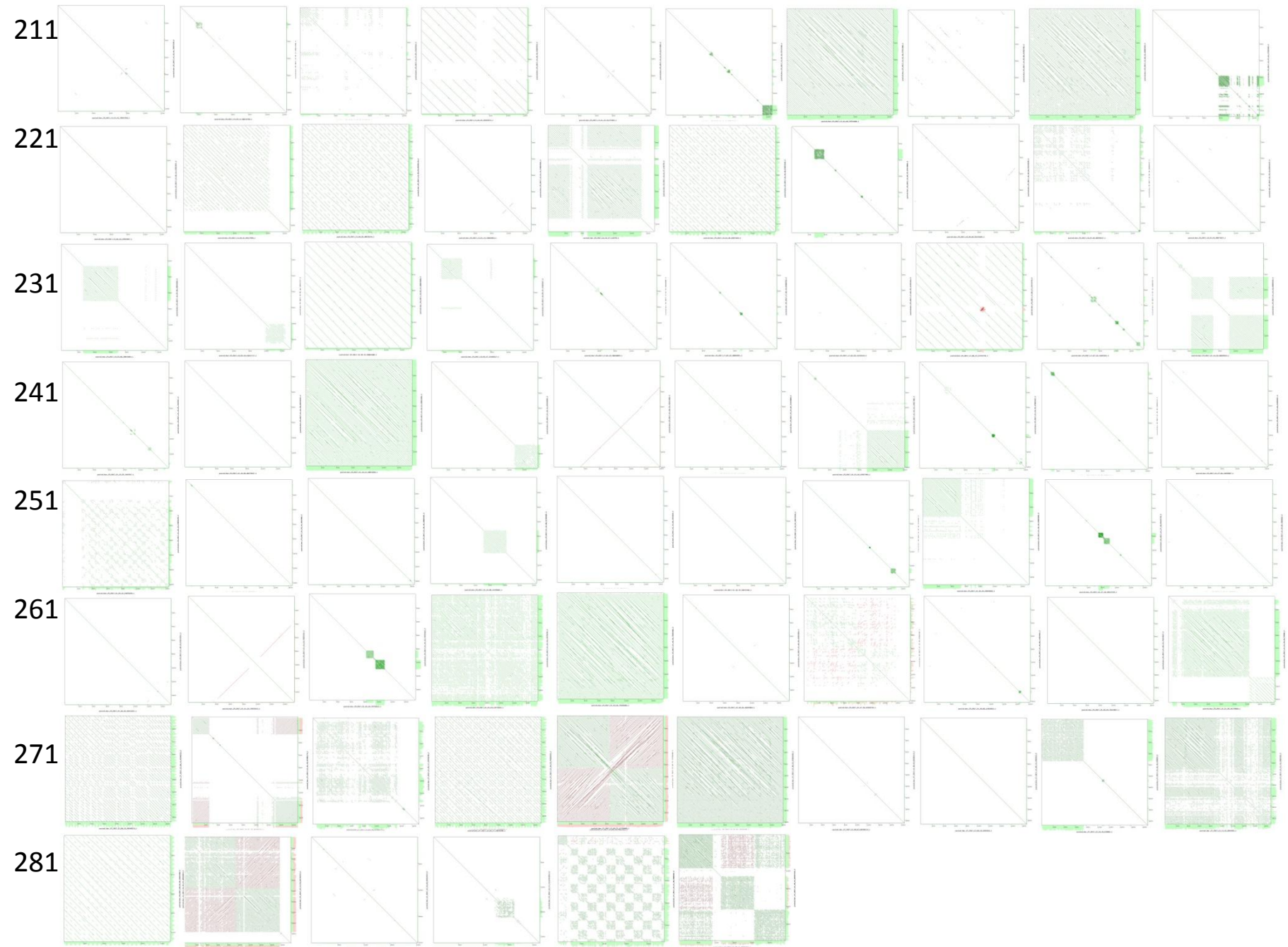

Supplement: Supplementary file 6 — Dot plot diagrams resulting from self to self comparison of long PaBio reads. (PDF 1371 kb) [file 12864_2017_3774_MOESM6_ESM.pdf]
