# Supplementary material for: Higher-order organisation of extremely amplified, potentially functional and massively methylated 5S rDNA in European pikes (Esox sp.)
Source: BMC Genomics. 2017 May 18;18:391. doi: 10.1186/s12864-017-3774-7 (PMC5437419; doi:10.1186/s12864-017-3774-7)
Supplement: Supplementary file 4 — Type, position, frequency and coverage of 5S rDNA polymorphisms. Table S1 – Analysis of genomic Illumina reads. Table S2 – Analysis of transcriptomic Illumina reads. SNPs along whole 5S units (genic and the intergenic spacer) were analyzed using genomic reads; for the transcriptome, only the genic region was considered. Note, high number of SNPs in spacer region compared to the genic region despite the shorter length (Table S1). SNP – single nucleotide polymorphism; MNP – multiple nucleotide polymorphism. (PDF 870 kb) [file 12864_2017_3774_MOESM4_ESM.pdf]

Tables S1 and S2. Type, position, frequency and coverage of 5S rDNA polymorphisms. Table S1 – Analysis of genomic Illumina reads. Table S2 – Analysis of transcriptomic Illumina reads. SNPs along whole 5S units (genic and the intergenic spacer) were analyzed using genomic reads; for the transcriptome, only the genic region was considered. Note, high number of SNPs in spacer region compared to the genic region despite the shorter length (Table S1). SNP – single nucleotide polymorphism; MNP – multiple nucleotide polymorphism.

Table S1. Distribution and frequency of polymorphisms in 5S rDNA

Region	Position	Type	Reference	Allele	Length	Count	Coverage	Frequency
Coding	16..17	MNP	TC	AG	2	4166	67173	6,201896595
Coding	19^20	Insertion	T	-	1	3757	67775	5,54334194
Coding	45	SNP	C	T	1	20206	184507	10,95134602
Spacer	129	SNP	G	A	1	73712	260028	28,3477164
Spacer	137	SNP	T	G	1	20440	268247	7,619842906
Spacer	139	SNP	A	T	1	25871	268247	9,644469463
Spacer	139^140	Insertion	-	T	1	79969	258107	30,98288694
Spacer	143	SNP	G	T	1	46921	257975	18,18819653
Spacer	143^144	Insertion	-	T	1	58323	253790	22,98081091
Spacer	145	SNP	A	G	1	62102	257496	24,1176562
Spacer	152..155	MNP	TCCT	AGGA	4	44635	263333	16,95002146
Spacer	164	SNP	T	A	1	27145	278661	9,741226795
Spacer	191	SNP	G	A	1	15735	269645	5,835450314
Spacer	195	SNP	A	C	1	35943	270579	13,28373599
Spacer	212	SNP	T	A	1	22191	216043	10,27156631
Spacer	218	SNP	T	C	1	27548	193085	14,26729161

Source data:Illumina whole genome library
Accession: SRR1197513
Legend:
SNP single nucleotide polymorphism
MNP multiple nucleotide polymorphism

Table S2. Analysis of transcriptomic Illumina reads.

Region	Position	Type	Refere nce	Allele	Length	Count	Coverage	Frequency
Coding	45	SNP	C	T	1	43	123	34.96

Source data: Illumina cDNA

	Accession
Pooled transcriptoms	SRR1228711
	SRR1228710
	SRR1228725
	SRR1228712
	SRR1228729
