# Supplementary material for: Higher-order organisation of extremely amplified, potentially functional and massively methylated 5S rDNA in European pikes (Esox sp.)
Source: BMC Genomics. 2017 May 18;18:391. doi: 10.1186/s12864-017-3774-7 (PMC5437419; doi:10.1186/s12864-017-3774-7)
Supplement: Supplementary file 5 — Phylogenetic NJ trees constructed from 500 aligned Illumina reads derived from the 5S genic part (A) and an intergenic spacer (B). Well supported branches (bootstrap >60%) are indicated by blue arrows. (C) A sequence of the 5S clone b from E. lucius (GenBank KX965717.1) with highlighted subregions used in the phylogenetic analysis. (PDF 856 kb) [file 12864_2017_3774_MOESM5_ESM.pdf]

Figure S4. Phylogenetic NJ trees constructed from 500 aligned Illumina reads derived from the 5S genic part (A) and an intergenic spacer (B). Well supported branches (bootstrap >60%) are indicated by blue arrows. (C) A sequence of the 5S clone b from *E. lucius* (GenBank KX965717.1) with highlighted subregions used in the phylogenetic analysis.

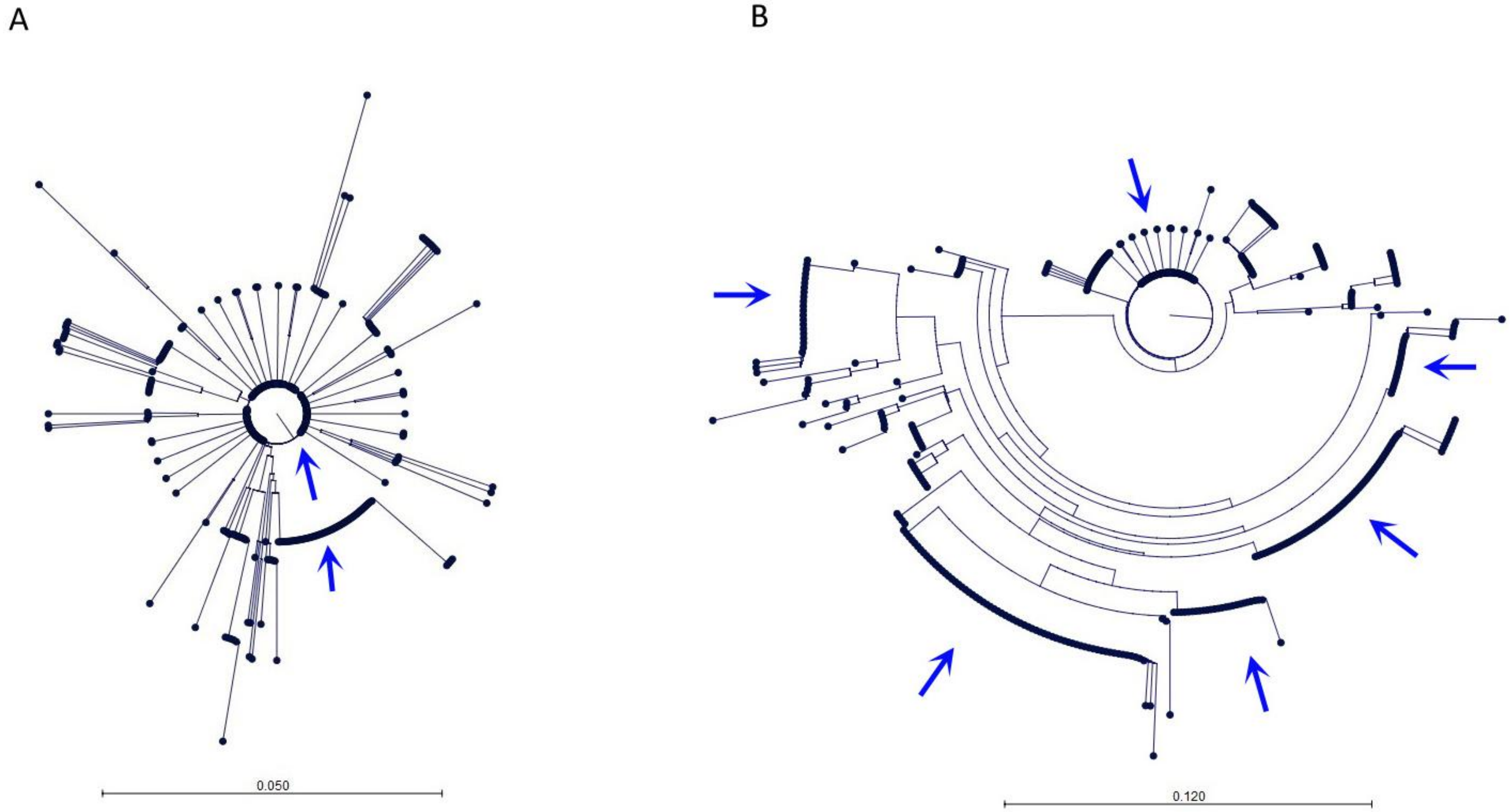

C

```
                   10        20        30        40        50        60
           ....|....|....|....|....|....|....|....|....|....|....|....|
KX965717.1 TAAGGCCGAATTTGGGCCGATTTTGGGAGGTAAACAAGGGTGGGCCCGGTTAGTAATTGG
                                                   IGS spacer subregion
                   70        80        90       100       110       120
           ....|....|....|....|....|....|....|....|....|....|....|....|
KX965717.1 ATGGGAGACCCACAAGGAATACCAGGGGCTGTTAAGCTTTTTTATTGGGGTCAATTTATT

                  130       140       150       160       170       180
|          ....|....|....|....|....|....|....|....|....|....|....|....|
KX965717.1 TTTGAAAAAAAAACCTGTCAATCATTTTTTTAGGACATGTCCACACACTTTATAAAAGGCC
                               5S genic subregion
                  190       200       210       220       230       240
           ....|....|....|....|....|....|....|....|....|....|....|....|
KX965717.1 CAAATTCAAACTGTCAATAGGGTTACGGCCAAACCCCCCTGAGCACGCCCGATCTTGTCC

                  250       260       270       280       290       300
           ....|....|....|....|....|....|....|....|....|....|....|....|
KX965717.1 GATCTCGGAAGCTAAGCAGGGTCGGGCCCGGTTAGTACTTGGATGGGAGACCGCCTGGGA

                  310       320       330       340       350       360
           ....|....|....|....|....|....|....|....|....|....|....|....|
KX965717.1 ATACCAGGTGCTGTAAGCCTTTTTGATTGGGGTCAATTAATTTGGAAAAAAATCCTGTCA

                  370       380       390       400       410       420
           ....|....|....|....|....|....|....|....|....|....|....|....|
KX965717.1 ATCATTCTTTTAGGACATGTCCACACATCTGTAAAGGCCCAAATACAAACTGCAAATTGG

                  430       440
           ....|....|....|....|....|.
KX965717.1 TTTACGGCCGTCCCACCCGGGAGGTC
```
