# Supplementary material for: Higher-order organisation of extremely amplified, potentially functional and massively methylated 5S rDNA in European pikes (Esox sp.)
Source: BMC Genomics. 2017 May 18;18:391. doi: 10.1186/s12864-017-3774-7 (PMC5437419; doi:10.1186/s12864-017-3774-7)
Supplement: Supplementary file 7 — Analysis of higher-order repeat structure of 5S rDNA using long (≥10 kb) PacBio reads. The selected sequences are ordered according to lengths (descending). Number of gene copies in reads was determined by MultiBlast. Arrangement was assessed by visual inspection of dot plot matrices (Additional file 6: Figure S5). Grouping followed the nomenclature in Table 2. Intergenic spacer variants: S–short (95–116 bp); L–long (321–340 bp) and UL–ultralong (1153–1209 bp). (PDF 471 kb) [file 12864_2017_3774_MOESM7_ESM.pdf]

Table S3. Analysis of higher-order repeat structure of 5S rDNA using long (≥10 kb) PacBio reads

The selected sequences are ordered according to lengths (descending). Number of gene copies in reads was determined by MultiBlast. Arrangement was assessed by visual inspection of dot plot matrices (Additional file 6: Figure S5). Grouping followed the nomenclature in Table 2. Intergenic spacer variants: S- short (95-116 bp); L - long (321-340 bp) and UL - ultralong (1153-1209 bp).

Read name	Read length	Number of genes	Arrangment	Spacer	Note
SRR1930096.496426 496426/1	29914	110	III	S	
SRR1930096.272159 272159/1	23963	78	III	S	
SRR1930096.5530 5530/1	23201	27	III	L	
SRR1930096.170906 170906/1	21471	3	IV	-	Contains a 18S rRNA gene
SRR1930096.109207 109207/1	20227	1	IV	-	
SRR1930096.499258 499258/1	19900	80	III	S	
SRR1930096.212523 212523/1	19210	82	II	S	
SRR1930096.531194 531194/1	19172	84	I	S	
SRR1930096.502520 502520/1	19041	85	I	S	
SRR1930096.311231 311231/1	18904	41	I	S	
SRR1930096.236717 236717/1	18777	2	IV	-	
SRR1930096.517944 517944/1	18569	1	IV	-	Contains 18S rRNA genes (2 copies)
SRR1930096.137860 137860/1	18311	82	I	S	
SRR1930096.248511 248511/1	18051	79	I	S	
SRR1930096.365967 365967/1	17676	79	III	S	
SRR1930096.29711 29711/1	17284	37	I	L	
SRR1930096.389394 389394/1	17118	54	II	S	
SRR1930096.216854 216854/1	17058	1	IV	-	
SRR1930096.296696 296696/1	16986	37	III	S	
SRR1930096.128907 128907/1	16884	35	I	S	
SRR1930096.237425 237425/1	16588	51	I	S	
SRR1930096.372549 372549/1	16569	2	IV	-	
SRR1930096.49823 49823/1	16372	5	IV	-	
SRR1930096.512607 512607/1	16246	2	IV	-	
SRR1930096.221130 221130/1	16167	56	III	S	

SRR1930096.143778 143778/1	15901	60	I	S	
SRR1930096.309549 309549/1	15823	38	I	S	
SRR1930096.23741 23741/1	15780	2	IV	-	
SRR1930096.360770 360770/1	15763	70	I	S	
SRR1930096.271579 271579/1	15737	64	I	S	
SRR1930096.213487 213487/1	15662	3	IV	-	
SRR1930096.197036 197036/1	15631	4	IV	-	
SRR1930096.5631 5631/1	15630	34	I	L	
SRR1930096.137861 137861/1	15625	70	I	S	
SRR1930096.201513 201513/1	15467	60	I	S	
SRR1930096.534279 534279/1	15463	1	IV	-	
SRR1930096.188980 188980/1	15336	67	I	S	
SRR1930096.212522 212522/1	15318	70	I	S	
SRR1930096.433067 433067/1	15262	4	IV	-	
SRR1930096.364733 364733/1	15192	2	IV	-	
SRR1930096.362652 362652/1	15181	35	II	S	
SRR1930096.243699 243699/1	15160	42	II	S	
SRR1930096.438306 438306/1	15143	28	II	S	
SRR1930096.230132 230132/1	15135	1	IV	-	
SRR1930096.187185 187185/1	14894	1	IV	-	
SRR1930096.209699 209699/1	14708	58	II	S	
SRR1930096.176236 176236/1	14697	68	I	S	
SRR1930096.240505 240505/1	14633	1	IV	-	
SRR1930096.400051 400051/1	14602	15	II	S	
SRR1930096.209074 209074/1	14577	1	IV	-	
SRR1930096.216183 216183/1	14493	2	IV	-	
SRR1930096.478921 478921/1	14424	46	III	S	
SRR1930096.293595 293595/1	14403	2	IV	-	
SRR1930096.136408 136408/1	14385	68	I	S	
SRR1930096.108844 108844/1	14299	9	II	S	
SRR1930096.380190 380190/1	14292	1	IV	-	
SRR1930096.400109 400109/1	14243	4	IV	-	
SRR1930096.304374 304374/1	14218	35	II	S	Contains 5S-unrelated tandem repeat

SRR1930096.210588 210588/1	14205	35	II	S	
SRR1930096.370793 370793/1	14170	1	IV	-	
SRR1930096.24612 24612/1	14068	1	IV	-	Palindrome of a unique DNA
SRR1930096.345167 345167/1	13978	15	II	S	
SRR1930096.548451 548451/1	13962	62	I	S	
SRR1930096.248512 248512/1	13952	63	I	S	
SRR1930096.204219 204219/1	13940	4	IV	-	
SRR1930096.505011 505011/1	13940	1	IV	-	
SRR1930096.136409 136409/1	13926	63	I	S	
SRR1930096.263669 263669/1	13856	34	II	S	
SRR1930096.199088 199088/1	13791	2	IV	-	
SRR1930096.422945 422945/1	13779	11	I	UL	
SRR1930096.171625 171625/1	13710	3	IV	-	
SRR1930096.58887 58887/1	13672	63	I	S	
SRR1930096.426241 426241/1	13542	49	II	S	
SRR1930096.432837 432837/1	13478	1	IV	-	
SRR1930096.414263 414263/1	13436	2	IV	-	
SRR1930096.436974 436974/1	13435	56	II	S	
SRR1930096.453218 453218/1	13368	14	II	S	
SRR1930096.392764 392764/1	13363	12	I	UL	
SRR1930096.11137 11137/1	13328	17	II	L	
SRR1930096.511013 511013/1	13319	51	III	S	
SRR1930096.202968 202968/1	13315	12	II	S	
SRR1930096.176237 176237/1	13304	63	I	S	
SRR1930096.496446 496446/1	13281	22	II	S	
SRR1930096.445537 445537/1	13247	1	IV	-	
SRR1930096.148522 148522/1	13156	12	II	S	
SRR1930096.238348 238348/1	13136	35	II	S	
SRR1930096.334366 334366/1	13069	3	IV	-	
SRR1930096.164059 164059/1	13046	2	IV	-	
SRR1930096.421211 421211/1	13043	51	II	S	
SRR1930096.11712 11712/1	13038	25	I	L	
SRR1930096.246063 246063/1	12997	46	II	S	

SRR1930096.459714 459714/1	12943	1	IV	-
SRR1930096.350995 350995/1	12927	39	II	S
SRR1930096.335531 335531/1	12880	62	I	S
SRR1930096.11713 11713/1	12861	27	I	L
SRR1930096.151631 151631/1	12847	3	IV	-
SRR1930096.532279 532279/1	12796	58	I	S
SRR1930096.208353 208353/1	12788	1	IV	-
SRR1930096.206397 206397/1	12756	1	IV	-
SRR1930096.470305 470305/1	12749	58	I	S
SRR1930096.432758 432758/1	12720	56	I	S
SRR1930096.203563 203563/1	12703	2	IV	-
SRR1930096.478659 478659/1	12688	54	I	S
SRR1930096.521836 521836/1	12688	57	I	S
SRR1930096.124138 124138/1	12672	25	I	L
SRR1930096.507555 507555/1	12670	1	IV	S
SRR1930096.39018 39018/1	12626	1	IV	S
SRR1930096.190845 190845/1	12624	60	I	S
SRR1930096.386425 386425/1	12501	8	I	UL
SRR1930096.537137 537137/1	12490	26	II	S
SRR1930096.408756 408756/1	12448	1	IV	-
SRR1930096.39020 39020/1	12427	1	IV	-
SRR1930096.39019 39019/1	12401	1	IV	-
SRR1930096.411007 411007/1	12379	52	II	S
SRR1930096.128607 128607/1	12321	19	I	L
SRR1930096.398710 398710/1	12314	55	II	S
SRR1930096.148925 148925/1	12251	53	I	S
SRR1930096.125806 125806/1	12239	11	II	S
SRR1930096.461230 461230/1	12231	44	II	S
SRR1930096.281235 281235/1	12196	52	I	S
SRR1930096.477383 477383/1	12168	27	II	S
SRR1930096.148927 148927/1	12132	53	I	S
SRR1930096.464633 464633/1	12132	54	I	S
SRR1930096.239970 239970/1	12130	56	I	S

SRR1930096.354940 354940/1	12093	57	I	S	
SRR1930096.119609 119609/1	12075	25	I	L	
SRR1930096.77416 77416/1	12072	22	II	S	
SRR1930096.18806 18806/1	12057	2	IV	-	
SRR1930096.382910 382910/1	12051	54	I	S	
SRR1930096.500259 500259/1	12046	42	II	S	Highly degenerated arrays
SRR1930096.342599 342599/1	12007	45	II	S	
SRR1930096.56196 56196/1	11994	55	II	S	
SRR1930096.148926 148926/1	11977	53	I	S	
SRR1930096.318517 318517/1	11964	7	II	S	
SRR1930096.412383 412383/1	11934	51	II	S	
SRR1930096.246307 246307/1	11928	41	I	S	
SRR1930096.299987 299987/1	11919	51	I	S	
SRR1930096.467414 467414/1	11890	20	II	S	
SRR1930096.379594 379594/1	11888	54	I	S	
SRR1930096.417513 417513/1	11871	8	IV	-	
SRR1930096.228492 228492/1	11865	1	IV	-	
SRR1930096.9847 9847/1	11862	4	II	L	
SRR1930096.120258 120258/1	11837	22	I	L	
SRR1930096.540651 540651/1	11820	5	IV	-	
SRR1930096.107916 107916/1	11711	4	IV	-	
SRR1930096.422049 422049/1	11705	1	IV	-	
SRR1930096.274325 274325/1	11681	7	I	UL	
SRR1930096.67498 67498/1	11635	53	I	S	
SRR1930096.204121 204121/1	11634	55	I	S	
SRR1930096.54293 54293/1	11628	3	IV	-	
SRR1930096.261496 261496/1	11622	23	II	S	
SRR1930096.419215 419215/1	11584	53	I	S	
SRR1930096.116578 116578/1	11565	5	II	S	
SRR1930096.216311 216311/1	11532	42	II	S	
SRR1930096.343219 343219/1	11510	43	II	S	
SRR1930096.491115 491115/1	11497	52	I	S	
SRR1930096.268893 268893/1	11472	1	IV	-	

SRR1930096.374737 374737/1	11462	53	I	S	
SRR1930096.319099 319099/1	11448	53	I	S	
SRR1930096.399287 399287/1	11432	51	I	S	
SRR1930096.549783 549783/1	11412	5	II	UL	
SRR1930096.288035 288035/1	11396	32	II	S	
SRR1930096.258145 258145/1	11374	47	I	S	
SRR1930096.248847 248847/1	11358	48	I	S	
SRR1930096.245783 245783/1	11350	1	IV	-	
SRR1930096.274326 274326/1	11335	8	I	UL	
SRR1930096.549784 549784/1	11334	5	II	UL	
SRR1930096.274327 274327/1	11326	9	I	UL	
SRR1930096.228033 228033/1	11323	3	IV	-	
SRR1930096.540264 540264/1	11304	20	II	S	
SRR1930096.258232 258232/1	11290	11	IV	-	
SRR1930096.49437 49437/1	11268	1	IV	-	
SRR1930096.350994 350994/1	11258	47	II	S	
SRR1930096.300705 300705/1	11253	47	I	S	
SRR1930096.296745 296745/1	11245	48	I	S	
SRR1930096.181159 181159/1	11199	1	IV	-	
SRR1930096.229409 229409/1	11155	9	IV	-	
SRR1930096.297800 297800/1	11151	51	I	S	
SRR1930096.293877 293877/1	11143	1	IV	-	
SRR1930096.17209 17209/1	11140	24	I	L	
SRR1930096.402937 402937/1	11131	2	IV	-	
SRR1930096.378832 378832/1	11126	1	IV	-	
SRR1930096.220742 220742/1	11120	36	II	S	
SRR1930096.72400 72400/1	11090	42	I	S	
SRR1930096.302654 302654/1	11054	29	III	S	
SRR1930096.300706 300706/1	11049	40	I	S/L	Both spacer variants present
SRR1930096.128527 128527/1	11048	24	I	L	
SRR1930096.323192 323192/1	11021	2	IV	-	
SRR1930096.437475 437475/1	11001	4	IV	-	
SRR1930096.501049 501049/1	10989	46	II	S	

SRR1930096.370702 370702/1	10974	25	II	S
SRR1930096.334367 334367/1	10968	2	IV	-
SRR1930096.509943 509943/1	10967	48	I	S
SRR1930096.524458 524458/1	10963	27	II	S
SRR1930096.160813 160813/1	10948	31	I	S
SRR1930096.271174 271174/1	10931	49	I	S
SRR1930096.372973 372973/1	10929	51	I	S
SRR1930096.542045 542045/1	10894	48	I	S
SRR1930096.43602 43602/1	10877	46	II	S
SRR1930096.25641 25641/1	10875	3	IV	-
SRR1930096.240428 240428/1	10866	2	IV	-
SRR1930096.98077 98077/1	10862	3	IV	-
SRR1930096.542308 542308/1	10844	4	IV	-
SRR1930096.300696 300696/1	10797	6	IV	-
SRR1930096.490851 490851/1	10784	47	II	S
SRR1930096.500360 500360/1	10754	39	II	S
SRR1930096.137948 137948/1	10701	2	IV	-
SRR1930096.283857 283857/1	10697	48	I	S
SRR1930096.530971 530971/1	10684	1	IV	-
SRR1930096.539484 539484/1	10683	1	IV	-
SRR1930096.322534 322534/1	10672	1	IV	-
SRR1930096.431192 431192/1	10638	48	II	S
SRR1930096.158146 158146/1	10627	2	IV	-
SRR1930096.540765 540765/1	10626	4	II	S
SRR1930096.477382 477382/1	10609	26	II	S
SRR1930096.232398 232398/1	10594	51	I	S
SRR1930096.233776 233776/1	10579	37	II	S
SRR1930096.455773 455773/1	10563	1	IV	-
SRR1930096.264506 264506/1	10527	28	II	S
SRR1930096.383110 383110/1	10517	46	III	S
SRR1930096.542751 542751/1	10513	2	IV	-
SRR1930096.243802 243802/1	10510	50	I	S
SRR1930096.221930 221930/1	10488	35	II	S

SRR1930096.478609 478609/1	10478	7	IV	-
SRR1930096.342353 342353/1	10468	46	I	S
SRR1930096.504298 504298/1	10466	2	IV	-
SRR1930096.348278 348278/1	10446	4	IV	-
SRR1930096.553924 553924/1	10443	43	II	S
SRR1930096.314120 314120/1	10440	21	II	S
SRR1930096.527559 527559/1	10439	21	II	S
SRR1930096.401428 401428/1	10416	2	IV	-
SRR1930096.302081 302081/1	10403	45	II	S
SRR1930096.17135 17135/1	10380	2	IV	-
SRR1930096.163066 163066/1	10371	8	II	S
SRR1930096.452389 452389/1	10362	1	IV	-
SRR1930096.262967 262967/1	10361	51	I	S
SRR1930096.407903 407903/1	10359	1	IV	-
SRR1930096.131165 131165/1	10358	23	I	L
SRR1930096.379366 379366/1	10351	3	IV	-
SRR1930096.373336 373336/1	10338	2	IV	-
SRR1930096.103682 103682/1	10323	36	II	S
SRR1930096.549089 549089/1	10323	38	II	S
SRR1930096.466501 466501/1	10303	1	IV	-
SRR1930096.348324 348324/1	10302	54	II	S
SRR1930096.552914 552914/1	10296	45	I	S
SRR1930096.159217 159217/1	10264	7	IV	-
SRR1930096.509176 509176/1	10262	1	IV	-
SRR1930096.54238 54238/1	10259	46	I	S
SRR1930096.420121 420121/1	10256	5	II	S
SRR1930096.351216 351216/1	10254	2	IV	-
SRR1930096.412382 412382/1	10249	33	II	S
SRR1930096.431193 431193/1	10245	45	I	S
SRR1930096.257020 257020/1	10233	1	IV	-
SRR1930096.314891 314891/1	10210	1	IV	-
SRR1930096.105426 105426/1	10203	45	I	S
SRR1930096.43449 43449/1	10203	47	I	S

SRR1930096.265727 265727/1	10192	1	IV	-
SRR1930096.300707 300707/1	10191	46	I	S
SRR1930096.488338 488338/1	10175	2	IV	-
SRR1930096.501051 501051/1	10169	44	I	S
SRR1930096.367535 367535/1	10167	2	IV	-
SRR1930096.136975 136975/1	10161	44	I	S
SRR1930096.536832 536832/1	10156	1	IV	-
SRR1930096.70194 70194/1	10153	1	IV	-
SRR1930096.145226 145226/1	10133	2	IV	-
SRR1930096.397385 397385/1	10121	2	IV	-
SRR1930096.139409 139409/1	10119	3	IV	-
SRR1930096.509941 509941/1	10114	46	I	S
SRR1930096.280600 280600/1	10105	2	IV	-
SRR1930096.208140 208140/1	10103	2	IV	-
SRR1930096.186836 186836/1	10102	1	IV	-
SRR1930096.375233 375233/1	10102	44	I	S
SRR1930096.153874 153874/1	10089	6	IV	-
SRR1930096.532664 532664/1	10084	1	IV	-
SRR1930096.353902 353902/1	10082	28	IV	-
SRR1930096.552916 552916/1	10079	44	I	S
SRR1930096.332651 332651/1	10056	38	II	S
SRR1930096.1697 1697/1	10041	24	I	L
SRR1930096.503842 503842/1	10032	45	I	S
SRR1930096.536830 536830/1	10026	1	IV	-
SRR1930096.412256 412256/1	10017	44	I	S
SRR1930096.158147 158147/1	10014	2	IV	-
SRR1930096.100222 100222/1	10011	45	II	S
SRR1930096.462997 462997/1	10009	4	IV	-
SRR1930096.32196 32196/1	10007	5	IV	-
SRR1930096.507765 507765/1	10000	43	II	S
**Total**	**3590130**	**7769**		
