# Supplementary material for: Higher-order organisation of extremely amplified, potentially functional and massively methylated 5S rDNA in European pikes (Esox sp.)
Source: BMC Genomics. 2017 May 18;18:391. doi: 10.1186/s12864-017-3774-7 (PMC5437419; doi:10.1186/s12864-017-3774-7)

Figure S6. A group III molecule #499258 (19900 bp) organised in two large immediately linked inverted blocks of tandem repeats. Green and red slanted lines indicate direct and inverted orientation of units, respectively. (B) The junction region alignment to 5S. Note, absence of any 5S-unrelated sequence between inverted repeats. Note, a deletion of the spacer in the third copy. (C) Nucleotide sequence of the junction region with annotated coding sequence (brown) and intergenic spacers (green).

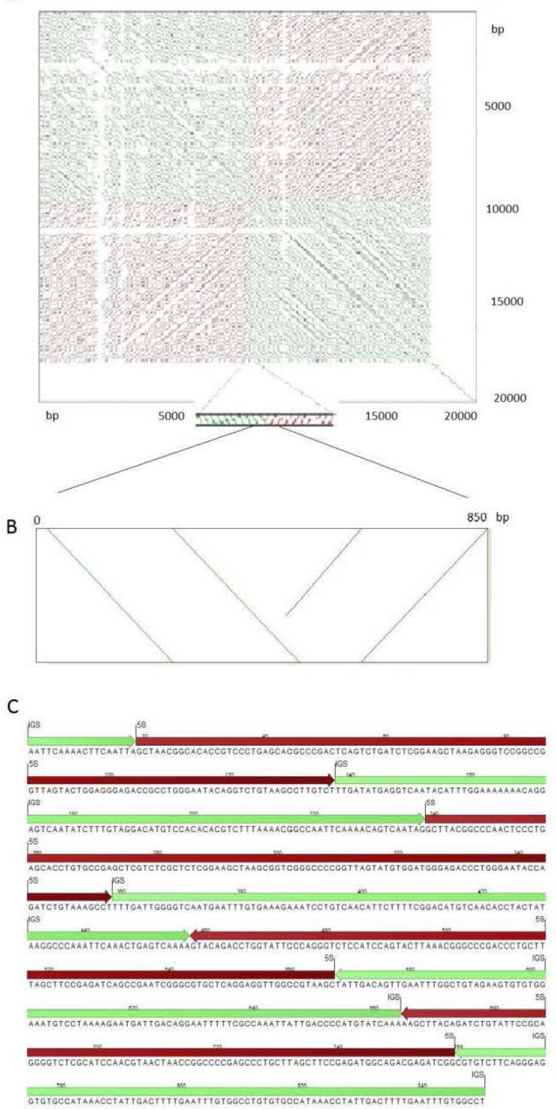

Supplement: Supplementary file 8 — A group III molecule #499258 (19,900 bp) organised in two large immediately linked inverted blocks of tandem repeats. Green and red slanted lines indicate direct and inverted orientation of units, respectively. (B) The junction region alignment to 5S. Note, absence of any 5S-unrelated sequence between the inverted repeats. Note, a partial deletion of IGS in the third copy. (C) Nucleotide sequence of the junction region with annotated genic sequence (brown) and IGS (green). (PDF 291 kb) [file 12864_2017_3774_MOESM8_ESM.pdf]
